# Phytochemicals from *Anneslea fragrans* Wall. and Their Hepatoprotective and Anti-Inflammatory Activities

**DOI:** 10.3390/molecules28145480

**Published:** 2023-07-18

**Authors:** Yan Wang, Changshu Cheng, Tianrui Zhao, Jianxin Cao, Yaping Liu, Yudan Wang, Wenbing Zhou, Guiguang Cheng

**Affiliations:** 1 Faculty of Food Science and Engineering, Kunming University of Science and Technology, Kunming 650500, China; 20212114040@stu.kust.edu.cn (Y.W.); chengchangshu0409@163.com (C.C.); food363@163.com (T.Z.); jxcao321@hotmail.com (J.C.); liuyaping@kust.edu.cn (Y.L.); 2 Key Laboratory of Chemistry in Ethnic Medicinal Resources, State Ethnic Affairs Commission and Ministry of Education, Yunnan Minzu University, Kunming 650500, China; sdlcwyd@163.com; 3 Yunnan Tobacco Company, Yuxi Branch, Yuxi 653100, China; yaowufx2001@163.com

**Keywords:** *Anneslea fragrans* Wall., phytochemical, isolation, hepatoprotective effect, anti-inflammatory activity

## Abstract

*Anneslea fragrans* Wall., popularly known as “Pangpo tea”, is an edible, medicinal, and ornamental plant of the Family Theaceae. The leaves of *A. fragrans* were historically applied for the treatment of liver and intestinal inflammatory diseases in China. This study aimed to explore the hepatoprotective agents from *A. fragrans* leaves through hepatoprotective and anti-inflammatory assessment. The phytochemical investigation of the leaves of *A. fragrans* resulted in the isolation and identification of a total of 18 chemical compounds, including triterpenoids, aliphatic alcohol, dihydrochalcones, chalcones, flavanols, phenolic glycoside, and lignans. Compounds **1**–**2**, **4**–**6**, **11**–**12**, and **16**–**18** were identified from *A. fragrans* for the first time. Compounds **7** and **14** could significantly alleviate hepatocellular damage by decreasing the contents of aspartate aminotransferase (AST) and alanine aminotransferase (ALT) and inhibit the hepatocellular apoptosis in the HepG2 cells induced by N-acetyl-p-aminophenol (APAP). In addition, compounds **7** and **14** inhibited reactive oxygen species (ROS) and malondialdehyde (MDA) contents and increased the catalase (CAT) superoxide dismutase (SOD), and glutathione (GSH) levels for suppressing APAP-induced oxidative stress. Additionally, compounds **7**, **13**, and **14** also had significant anti-inflammatory effects by inhibiting interleukin-6 (IL-6), interleukin-1β (IL-1β), and tumor necrosis factor-α (TNF-α) productions on LPS-induced RAW246.7 cells.

## 1. Introduction

As an important organ for the metabolism and detoxification of exogenous chemicals, the liver plays a vital role in the biotransformation of non-nutrients [1,2]. Excessive administration of exogenous substances, including viruses, drugs, and other chemicals, will induce the occurrence of oxidative stress in liver tissue [3], which will further induce liver inflammation, liver fibrosis, and liver cirrhosis [4,5], and eventually lead to hepatocellular carcinoma. The imbalance between the production and elimination of reactive oxygen species (ROS) is a critical factor in oxidative stress-induced liver injury. Therefore, inhibiting ROS accumulation might be a potential strategy in the prevention and treatment of liver injury.

Recently, many phytochemical antioxidants from edible and medicinal plants have been investigated to treat liver injury. These compounds had a significant potential capacity to scavenge the free radicals, inhibit lipid peroxidation, and promote the expressions of antioxidant enzymes systems, which included catalase (CAT), superoxide dismutase (SOD), and glutathione peroxidase (GSH-Px). To date, flavonoids, especially dihydrochalcones, are important natural antioxidants and have reported remarkable hepatoprotective effects for treating liver diseases. For example, neohesperidin dihydrochalcone (NHDC) has a significant hepatoprotective effect as it increases antioxidant enzyme activities to inhibit liver damage induced by oxidative stress in mice treated with CCl_4_ [6]. Thus, the search for natural hepatoprotective agents is urgent for patients suffering from liver diseases.

**Figure 1:**
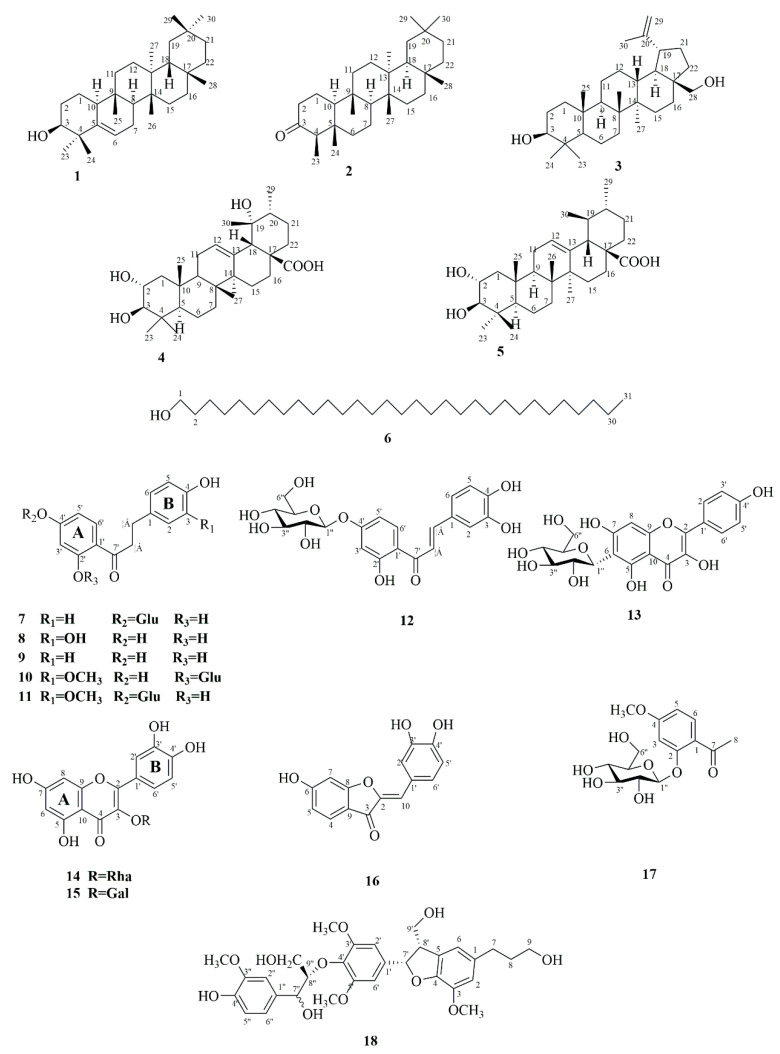
Structures of the isolated compounds from *A. fragrans*.

*Anneslea fragrans* Wall., an evergreen plant, is mainly distributed in Southern China [7]. It has been historically used to treat liver and intestinal inflammation diseases in “dai” ethno-pharmacy [8]. Traditionally, its leaves and bark are also used as food supplements in wine and made into folk tea beverages, known as “Pangpo tea” [9]. *A. fragrans* presented a number of phenolic compounds [10], including chalcones (fragranone B, and butein), dihydrochalcones (fragranone A, 3,4,2′,4′-tetrahydroxydihydrochalcone, confusoside, vacciniifolin, and davidioside), isoflavones (afzelin, quercitrin, isoquercitrin, kaempferol 3-neohesperidoside, and nictoflorin), and so on [11]. Meanwhile, confusoside was the major flavonoid from *A. fragrans* (Figure 1). Some of them had a lot of biological activities, including antioxidant and anti-inflammatory activities [12]. In addition, the extract of *A. fragrans* leaves could alleviate ulcerative colitis (UC) induced by dextran sodium sulfate (DSS) in mice through suppressing NF-κB and MAPK pathway activation and improving intestinal barrier integrity [8]. However, there is no study on the hepatoprotective effect of *A. fragrans* and their biological agents. Thus, to fill that gap, this study aimed to isolate *A. fragrans* bioactive agents and explore their hepatoprotective effects on APAP-induced HepG2 cells and anti-inflammatory activities on LPS-induced RAW264.7 cells in vitro, respectively.

## 2. Results and Discussion

### 2.1. Identification of Phytochemicals from A. fragrans

Phytochemical investigation on the ethyl acetate extract from *A. fragrans* leaves (AFEA) resulted in the isolation of 18 compounds (**1**–**18**). The structures of isolated compounds were characterized using various spectroscopic techniques, such as high-resolution electrospray ionization mass spectroscopy (HRESIMS), nuclear magnetic resonance (NMR), and so on. These isolated compounds were determined as glutinol (**1**) [13], friedelin (**2**) [14], betulin (**3**) [15], 2α,19α-dihydroxyursolic acid (**4**) [16], 2α-hydroxyursolic acid (**5**) [17], 1-hentriacontanol (**6**) [18], confusoside (**7**) [19], 2′,3,4,4′-tetrahydroxydihydrochalcone (**8**) [20], 2′,4,4′-trihydroxydihydrochalcone (**9**) [21], fragranone C (**10**) [22], 1-[4-(β-d-glucopyranosyloxy)-2-hydroxyphenyl]-3-(4-hydroxy-3-methoxyphenyl)-1-propanone (**11**) [9], butein-4′-*O*-β-d-glucoside (**12**) [23], kaempferol 6-C-β-d-glucopyranoside (**13**) [24], quercetin-3-*O*-rhamnopyranoside (**14**) [25], quercetin-3-*O*-β-d-galactoside (**15**) [26], sulfuretin (**16**) [27], paeonoside (**17**) [28], and acernikol (**18**) [29]. According to the structural skeletons, the isolated compounds could be classified as triterpenoids (**1**–**5**), aliphatic alcohol (**6**), dihydrochalcones (**7**–**11**), chalcone (**12**), flavanols (**13**–**15**), aurone (**16**), phenolic glycoside (**17**), and lignan (**18**) (Figure 1). Among them, 10 compounds (**1**–**2**, **4**–**6**, **11**–**12**, and **16**–**18**) were first isolated from *A. fragrans*.

### 2.2. Hepatoprotective Effects of the Isolated Compounds on APAP-Induced HepG2 Cells

#### 2.2.1. Cytotoxic Activities of the Isolated Compounds on HepG2 Cells

As a human hepatoma cell line, HepG2 cells retain many special functions that can characterize normal liver cells and are generally considered as a good system for searching bioactive compounds [30]. Acetaminophen (APAP) is a common antipyretic and analgesic drug. However, it can produce a toxic intermediate metabolite NAPQI, which could be combined with GSH for detoxification. Furthermore, excessive administration of APAP may deplete GSH, thereby leading to the occurrence of oxidative stress [31]. Therefore, the APAP-induced oxidative stress in HepG2 cells is used as a common model in vitro to discover hepatoprotective agents.

Firstly, the MTT assay was used to determine the cytotoxicity of the isolated compounds on HepG2 cells. As shown in Supplementary Table S1, compounds **1**–**2**, **4**–**8**, and **10**–**18** had no toxicity to HepG2 cells at all the tested concentrations. However, compounds **3** and **9** had toxicity in the concentration of 150 μM with a cell survival rate of 88.36 ± 2.41 and 79.45 ± 0.89%, respectively. Therefore, the concentration of 150 μM was chosen for compounds **1**–**2**, **4**–**8**, and **10**–**18**, and 100 μM was chosen for compounds **3** and **9** to detect their protective effects on APAP-induced HepG2 cells.

**Figure 2:**
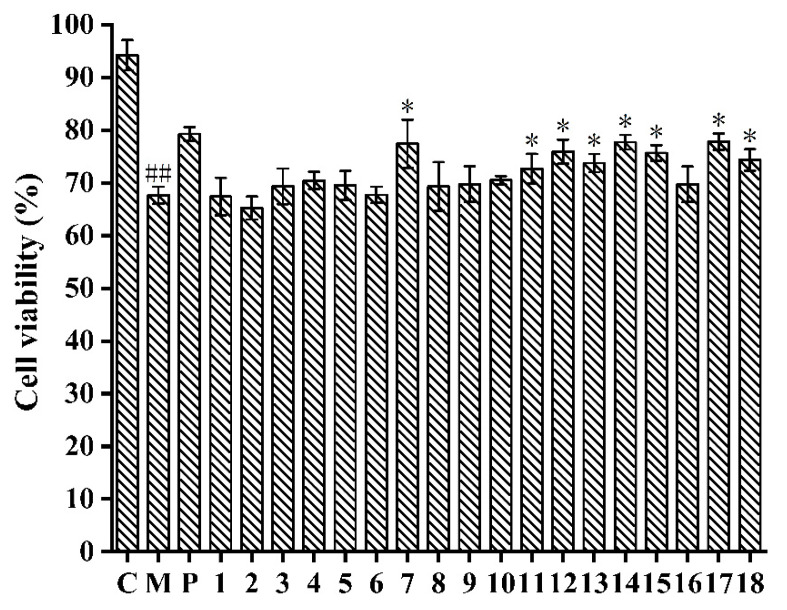
Compounds isolated from *A. fragrans* inhibited the APAP (10 mM)-induced hepatotoxicity in HepG2 cells. ^##^ *p* < 0.05 vs. group C; * *p* < 0.05 vs. group M; C: normal control group; M: acetaminophen (APAP) model group; P: N-Acetylcysteine (NAC) group.

In comparison with the control group (94.21 ± 2.87%), HepG2 cells treated with APAP (67.65 ± 1.62%) had lower cell viability (Figure 2), which indicated that the APAP-induced HepG2 cell injury model was successfully established [32]. Furthermore, the cell viability of APAP-induced HepG2 cells treated with compounds **7**, **11**–**15**, and **17**–**18** was significantly enhanced in comparison with the model group (*p* < 0.05). Particularly, compound **17** showed the strongest inhibitory effect against APAP-induced cell injury by increasing the viability of HepG2 cells from 67.65 ± 1.62% to 77.82 ± 1.54% at 150 μM, which exhibited a similar effect as the N-Acetylcysteine (NAC) (79.27 ± 1.32%). The isolated flavonoids (**7**, **11**–**15**) showed better hepatoprotective effects than triterpenoids (**1**–**5**) and aliphatic alcohol (**6**). Therefore, eight compounds (**7**, **11**–**15,** and **17**–**18**) were selected so we could determine their hepatoprotective effects against APAP-induced HepG2 cells.

#### 2.2.2. Inhibitory Effects of Isolated Compounds on ALT and AST Contents in APAP-Induced HepG2 Cells

Aspartate aminotransferase (AST) and alanine aminotransferase (ALT) are important biological indexes of liver injury, and their contents are closely related to the severity degree [33]. The protective effects of compounds **7**, **11**–**15,** and **17**–**18** on HepG2 cells induced by APAP were determined by measuring the ALT and AST contents.

**Figure 3:**
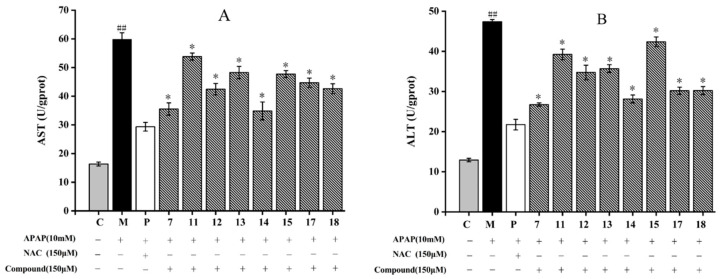
Effect of eight compounds isolated from *A. fragrans* on aspartate aminotransferase (AST) (**A**) and alanine aminotransferase (ALT) (**B**) levels in APAP-induced HepG2 cells. ^##^ *p* < 0.05 vs. group C; * *p* < 0.05 vs. group M (APAP group); C: normal control group; P: N-Acetylcysteine (NAC) group.

All the isolated compounds significantly diminished AST and ALT contents compared to those of the model group (*p* < 0.05) (Figure 3). For AST activity, among the eight compounds, compound **11** showed the weakest inhibitory effect with a level of AST 53.83 ± 1.23 U/gprot. In addition, compared to the model group, compound **14** showed the most inhibitory effect by decreasing the content of AST from 59.81 ± 2.31 U/gprot to 34.85 ± 3.12 U/gprot (*p* < 0.05) (Figure 3A). As shown in Figure 3B, compound **7** (26.74 ± 0.42 U/gprot) showed the strongest inhibitory effect with the lowest ALT content. Compound **15** had the lower inhibitory effect with the level of ALT 42.37 ± 1.21 U/gprot. The ALT contents of HepG2 cells treated with compounds **17** and **18** were basically the same (30.2 ± 0.89 U/gprot). According to the structure skeletons, compounds **7** and **14** are flavonoids. In a previous study, the flavonoids isolated from sweet tea, such as phloretin and phlorizin, also showed a good protective effect on APAP-induced liver injury [34]. Our results are in accordance with the results of liver protection that have already been reported.

#### 2.2.3. Inhibitory Effects of Isolated Compounds against APAP-Induced HepG2 Cells Apoptosis

The inhibitory effect of cell apoptosis can be used to prevent the development of liver injury induced by APAP [35]. A previous study has reported that the reactions of intracellular ROS with some amino acids in DNA repair proteins could eventually induce cell apoptosis by leading to the fragmentation of genomic DNA [36]. According to the research on cell death mechanism, APAP can dose-dependently cause nuclear DNA fragmentation without necrosis [37]. The protective effects of isolated compounds against APAP-induced HepG2 cells were evaluated by detecting the cell apoptosis rate in this study.

**Figure 4:**
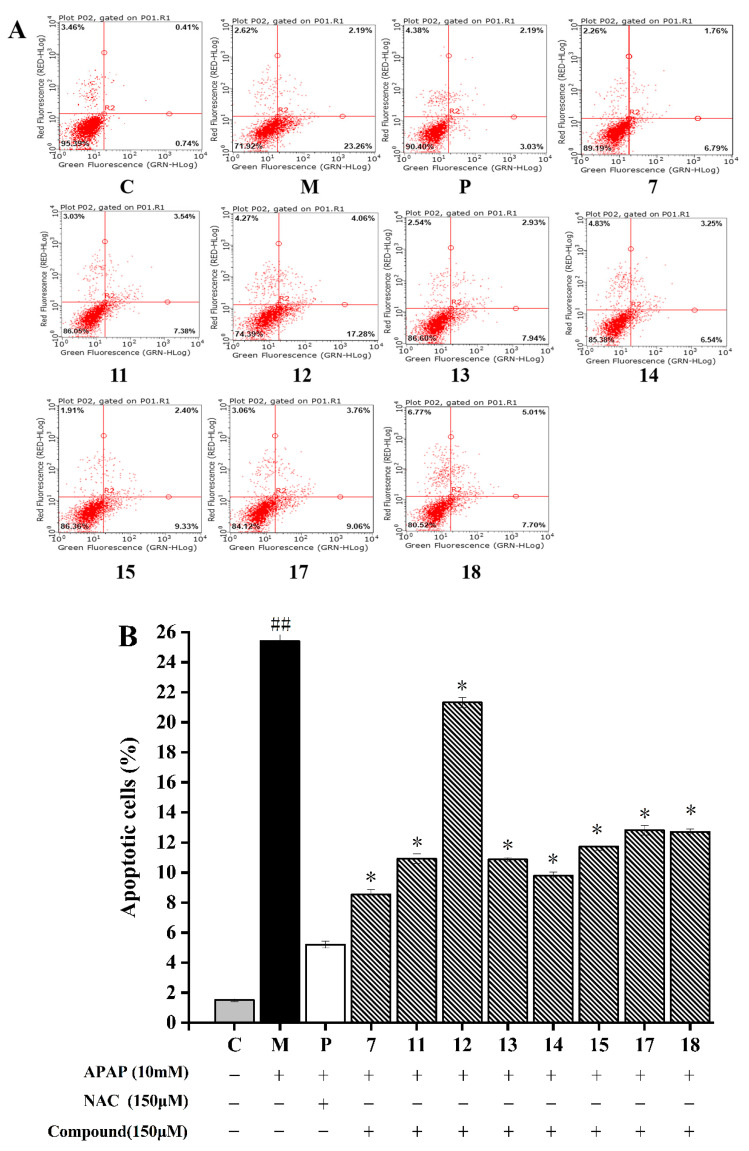
Effects of eight compounds isolated from *A. fragrans* on cell apoptosis. Flow cytometry analysis (**A**); the apoptotic cell percentage of different groups (**B**). ^##^ *p* < 0.05 vs. group C; * *p* < 0.05 vs. group M (APAP group); C: normal control group; P: N-Acetylcysteine (NAC) group.

The total apoptotic rate of HepG2 cells was composed of early apoptotic cells (lower right quadrant of Figure 4A) and late apoptotic cells (upper right quadrant of dot plot, Figure 4A). As described in Figure 4, HepG2 cells induced by the 10 mM APAP (25.41 ± 0.97%) exhibited a higher apoptosis rate than the normal cells (1.51 ± 0.04%) (*p* < 0.05). The cell apoptosis rate incubated with compounds **7**, **11**–**15**, and **17**–**18** significantly decreased in comparison to that treated with APAP (*p* < 0.05). Among these eight compounds, compound **7** had the strongest inhibitory effect on APAP-induced HepG2 cells by decreasing the apoptosis rate to 8.55 ± 0.31%, followed by compound **14** with apoptosis rate of 9.79 ± 0.26%. Compound **12** showed the weakest inhibitory effect with the rate of apoptosis 21.32 ± 0.33%. Generally, the anti-apoptosis activity of other compounds may be ordered as follows: compounds **11** = **13** > **15** > **17** = **18**. Thus, the flavonoids isolated from *A. fragrans* could effectively inhibit APAP-induced cell apoptosis.

#### 2.2.4. Inhibition of Isolated Compounds on Intracellular ROS Generation

ROS are oxygen-containing chemically reactive chemicals that performed as messengers in complex cellular processes, such as signal transduction, the regulation of cell proliferation, and gene expression [38]. However, excessive ROS production could lead to oxidative stress, mitochondrial dysfunction, and ultimately cell apoptosis or necrosis [39]. APAP can lead to an imbalance between ROS production and elimination, which eventually leads to oxidative stress [40]. In this study, the intracellular ROS scavenging capacity of the eight isolated compounds was determined on APAP-induced HepG2 cells.

**Figure 5:**
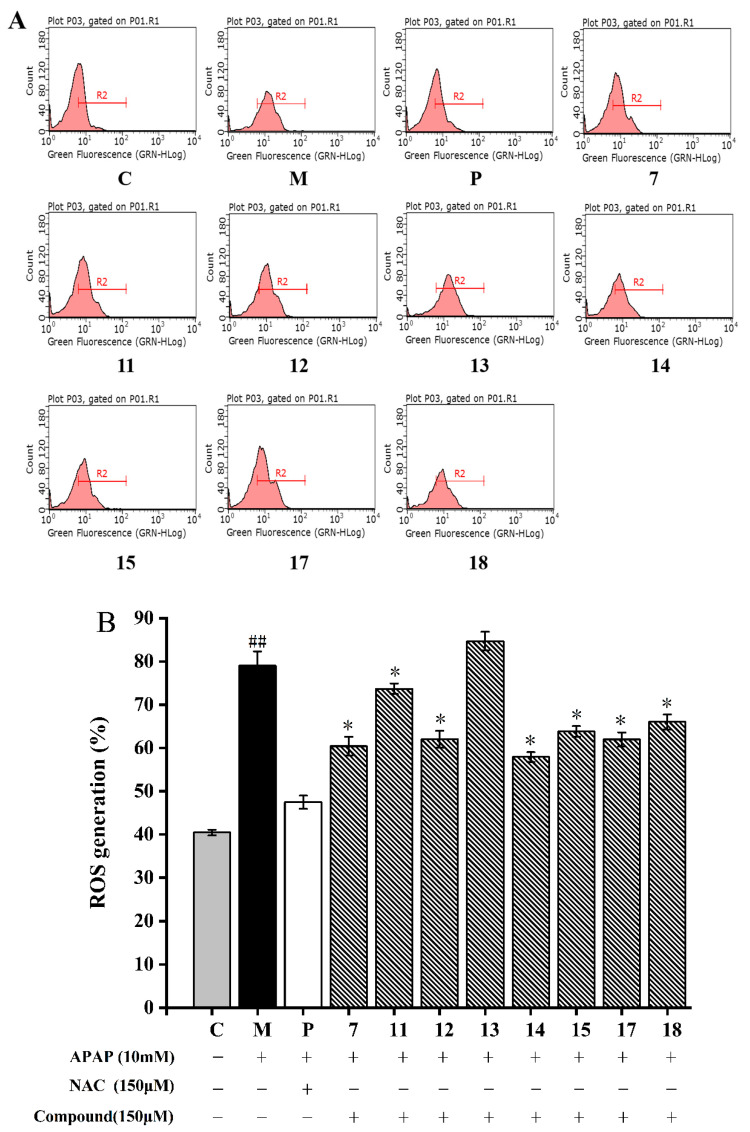
Cellular ROS inhibitory effects of eight compounds from *A. fragrans* in APAP-induced HepG2 cells. Flow cytometry analysis (**A**); quantitative analysis of ROS (**B**). ^##^ *p* < 0.05 vs. group C; * *p* < 0.05 vs. group M (APAP); C: normal control group; P: N-Acetylcysteine (NAC) group.

As shown in Figure 5A, the fluorescence signal of the model group was significantly shifted to the right *(p* < 0.05). After being treated with 10 mM APAP, the intracellular ROS content in the HepG2 cells increased to 79.03 ± 2.14%, which was almost two times higher than the normal cells (40.25 ± 0.67%) (*p* < 0.05, Figure 5). In addition, the right shift was significantly weakened by the treatment of compound **7**. Compounds **7**, **11**–**15**, and **17**–**18** significantly decreased the ROS levels in the HepG2 cells when compared to that treated with APAP (*p* < 0.05, Figure 5). Compound **14** showed the strongest ROS scavenging ability (53.22 ± 2.32%) on APAP-induced HepG2 cells, followed by compound **7**. The ROS scavenging activity of other compounds was found: **17** > **12** > **15** > **18** > **11** > **13**. This finding indicated that flavonoids in *A. fragrans* were good antioxidants, such as confusoside, and quercetin-3-*O*-rhamnopyranoside.

ROS is a general term for oxygen free radicals, such as superoxide anion (O_2_^−^), hydrogen peroxide (H_2_O_2_) and hydroxyl radical (OH•), which are highly oxidizing due to their unpaired electrons [41]. Studies reported that flavonoids have higher reducing power than other types of compounds (compounds **17** and **18**) due to their reducing groups, such as phenolic OH, and carbonyl groups [42]. As for compounds **14** and **15**, the 6″-OH group of compound **15** might form hydrogen bond interaction with H• radical due to the existence of two lone pair electrons, which effectively blocks the hydrogen supply ability of phenolic hydroxyl groups in ring A and ring B, and ultimately reduces the binding ability with OH• radical [41]. In comparison with compounds **14** and **15**, compound **13** exhibited a weaker antioxidant capacity, because the o-dihydroxyl group on the B ring of the compound **13** were more likely to form P-π conjugates and stable intramolecular hydrogen bonds, interrupting the chain reaction of free radicals (O_2_^−^) and obtaining better ability to scavenge ROS [43]. Furthermore, compared with compound **7**, compound **11** increased hydrophobicity and liposolubility due to the addition of a methoxy group, thereby reducing the binding ability to ROS, and the *ortho*-OH of compound **12** was easy to react with the α,β-unsaturated ketone system of the CH, which results in the phenolic hydroxyl group not being able to easily provide H^+^ to free radicals [44]. Conclusively, the flavonoids of *A. fragrans* with a strong ROS scavenging ability were depended on the hydrophilic phenolic hydroxyl groups and carbonyl groups.

#### 2.2.5. Effect of Isolated Compounds on Intracellular Antioxidant Enzymes in HepG2 Cells

The catalase (CAT) and superoxide dismutase (SOD) activities, and the contents of glutathion (GSH) and malondialdehyde (MDA) were measured to determine whether these eight compounds isolated from *A. fragrans* could improve the antioxidative enzyme system to inhibit APAP-induced oxidative stress in HepG2 cells.

**Figure 6:**
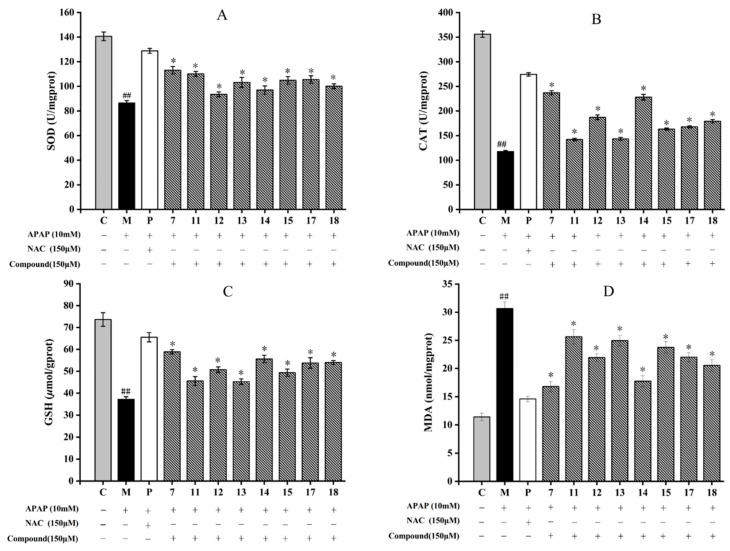
Effect of eight compounds of *A. fragrans* on superoxide dismutase (SOD) (**A**), catalase (CAT) (**B**), glutathione (GSH) (**C**), and malondialdehyde (MDA) (**D**) levels in APAP-induced HepG2 cells. ^##^ *p* < 0.05 vs. group C; * *p* < 0.05 vs. group M (APAP group); C: normal control group; P: N-Acetylcysteine (NAC) group.

The results showed that APAP could significantly inhibit the SOD and CAT activities, reduce the level of GSH and increase the MDA content in comparison with those of the control group (*p* < 0.05) (Figure 6D). Among eight compounds, compounds **7** and **14** all significantly increased SOD and CAT, and GSH levels in HepG2 cells (*p* < 0.05) (Figure 6A–C). Although compounds **11** and **13** had the lowest activity, their effects were still obvious in comparison with the model group (*p* < 0.05). Compounds **7** and **14** also obviously inhibited MDA production in HepG2 cells, while compounds **11**, **13**, and **15** had lower inhibitory effects (Figure 6D).

It is well known that O_2_**^−^** can be transferred to H_2_O_2_ and O_2_ with the catalyzation of SOD, and the H_2_O_2_ will be further metabolized by CAT to water and oxygen. Thereby, CAT and SOD are the main enzymes in the antioxidant defense system against oxidative stress [39]. MDA is the product of membrane lipid peroxidation induced by ROS, which are commonly known as indicators of oxidative stress [45]. GSH is an endogenous antioxidant component in plant tissues, which can effectively eliminate accumulations of ROS and MDA [39]. Studies had shown that flavonoids, such as kaempferol and quercetin, could up-regulate the activity of SOD and CAT enzymes and increase the content of GSH by activating the Nrf2 pathway [46,47]. Kaempferol and quercetin were the basic structures of compound **13**, and compounds **14** and **15**, respectively. The findings supported our results that the flavonoids isolated from *A. fragrans* could increase the activities of SOD and CAT, and the contents of GSH and MDA. Meanwhile, it was firstly to compare the antioxidant activities between the dihydrochalcones (compounds **7** and **11**), chalcone (compound **12**), and flavonols (compounds **13**–**15**).

#### 2.2.6. Multivariate Analysis

**Figure 7:**
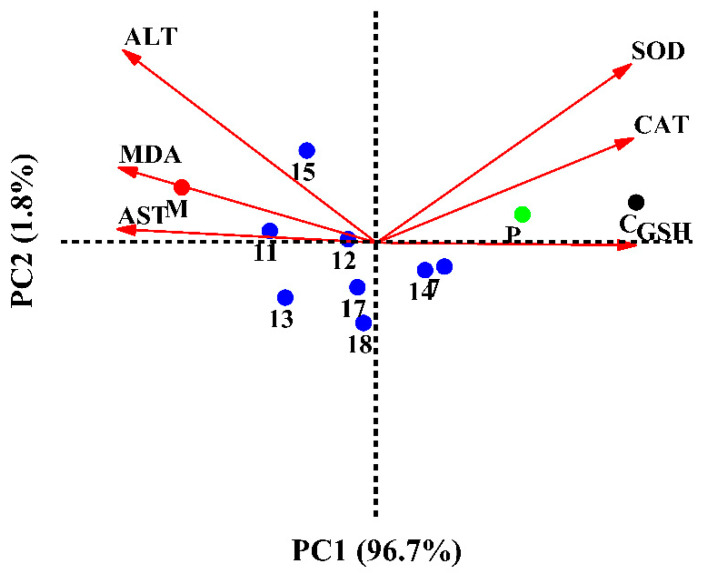
PCA analysis on cell protection and antioxidant activity.

Principal component analysis (PCA) was used to explain SOD, CAT, GSH, MDA, AST, and ALT mutation in HepG2 cells treated with the eight selected compounds. As shown in Figure 7, the total variation was explained to 98.50%, where PC1 accounted for 96.70% and PC2 for 1.8% of the variance. The just-right superior quadrant of PC1 included the control and positive (NAC) groups. Compounds **7** and **14** were involved in the straight lower right quadrant of PC1. HepG2 cells treated with compound **7** showed the highest content of GSH. Compounds **7** and **14** were characterized by high activities of SOD and CAT. Compound **15** had the lowest ALT scavenging activity and was located on the axis of PC2 with a negative score. Meanwhile, the consequences of compound **11** having the highest concentrations of MDA and AST were corresponding to the ROS and apoptosis results. As shown in Figure 7, the flavonoids had better hepatoprotective activities than the other type of compounds, such as phenolic glycoside (**17**) and lignan (**18**). Interestingly, the isolated flavonoids, such as confusoside (compound **7**) and quercetin-3-*O*-rhamnopyranoside (compound **14**), were the main antioxidants in *A. fragrans* leaves. Our data also showed that the hepatoprotective activities of different compounds in *A. fragrans* leaves were positively correlated with their antioxidant capacities.

### 2.3. The Inhibitory Effects of Isolated Compounds on Inflammatory Response on LPS Induced RAW264.7 Cells

#### 2.3.1. Inhibitory Effects of Isolated Compounds on NO Production

The inflammatory response is an important immune defense mechanism. In this response, RAW264.7 cells can produce pro-inflammatory cytokines in response to inflammatory stimuli [48]. The NO release from macrophages could be promoted by LPS, which was involved in septic shock [49]. NO can also interact with other free radicals to produce cytotoxic molecules. Therefore, inhibiting NO production is an anti-inflammatory treatment [50]. In the MTT assay, compounds **1**–**5**, **7**, **10**–**11,** and **13**–**18** exhibited no toxicity in all tested concentrations (Supplementary Table S2). However, compounds **6**, **8**, and **9** were toxic to the RAW264.7 cells at 100 μM concentration with the survival rates of 73.23 ± 1.54%, 85.85 ± 1.34%, and 71.95 ± 1.62%, respectively, while compound **12** exhibited toxicity to cells at 150 μM. Therefore, the maximum nontoxic dose was selected in the subsequent experiment.

Table 1 demonstrated that compounds **2**, **13**, and **14** had remarkable inhibitory effects on NO production with no significant difference compared with dexamethasone (DXM). After treatment of compound **13**, NO content was significantly decreased to 7.33 ± 0.52 μmol/gprot compared with the model group (13.65 ± 0.62 μmol/gprot) (*p* < 0.05). Compound **9** showed the weakest inhibitory effect of NO production with the content of NO 12.73 ± 0.37 μmol/gprot. In addition, compounds **15** and **17** had similar inhibitory effects on NO production. As shown in Table 1, the flavonoids had lower contents of NO compared with the other types of compounds. The data indicated that compounds **2**, **7**, **10**, **13**–**15**, and **17**–**18** possessed significantly inhibitory effects of NO in comparison with that of the model group (*p* < 0.05). Therefore, these eight compounds (**2**, **7**, **10**, **13**–**15**, and **17**–**18**) were selected to determine their inhibitory effects in pro-inflammatory cytokines, which included IL-6, IL-1β, and TNF-α.

#### 2.3.2. Inhibition of Related Inflammatory Factors

**Figure 8:**
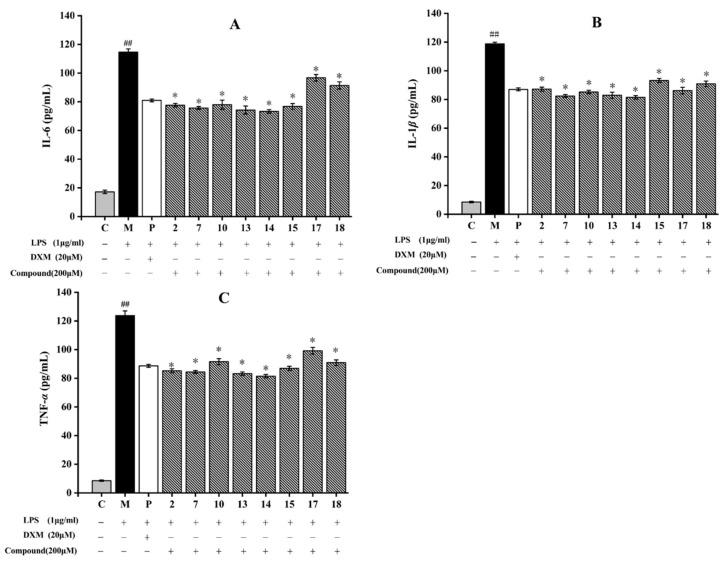
Effects of eight compounds isolated from *A. fragrans* on inflammatory cytokines levels including interleukin-6 (IL-6) (**A**), interleukin-1β (IL-1β) (**B**), and tumor necrosis factor-α (TNF-α) (**C**) in LPS-induced RAW264.7 cells. ^##^ *p* < 0.05 vs. group C; * *p* < 0.05 vs. group M (LPS group); C: normal control group; P: dexamethasone (DXM) group.

After induction by LPS, RAW264.7 cells can release high pro-inflammatory cytokines levels including IL-6, IL-1β, and TNF-α. They can resist pathogenic microorganism invasion, while excessive secretions will lead to inflammatory reactions [51]. Hence, the anti-inflammatory ability of eight compounds against RAW264.7 cells induced by LPS was determined. As shown in Figure 8, LPS treatment increased TNF-α, IL-1β, and IL-6 levels in the model group by comparison to those in the control group. Flavonoids (**7**, **13**–**15**) and triterpenoid (**2**) significantly decreased the production of IL-6 and TNF-α, and dihydrochalcone (**10**) dramatically suppress the release of IL-1β. Compounds **7**, **13**, and **14** had obvious inhibitory effects on these three inflammatory cytokines compared with those in the model group (*p* < 0.05). Compound **14**, a flavanol with an ortho-dihydroxyl moiety on the B-ring, showed the strongest inhibitory effect on LPS-induced IL-6, IL-1β, and TNF-α production among the tested compounds, and its inhibitory rates reached 36.3% (IL-6), 31.4% (IL-1β), and 34.2% (TNF-α) (Figure 8). Accumulation studies revealed that flavonoids, such as galangin and isobavachalcone, reduced the inflammatory expressions by suppressing NF-κB pathway, PI3K/Akt signaling pathway, and MAPK signaling pathway [52,53]. In this study, these flavonoids suppressed the inflammatory response by decreasing the inflammatory cytokines expressions, which may be related to these inflammatory pathways. Therefore, flavonoids compounds obtained from *A. fragrans* had effective anti-inflammatory effects, which was consistent with the previous study [54].

## 3. Materials and Methods

### 3.1. Chemicals and Reagents

The ultrapure water used was deionized using the Milli-Q system (Millipore, Bedford, MA, USA). Ethanol, dichloromethane, petroleum ether, and ethyl acetate were purchased from Tianjin Fuyu Fine Chemical Co., Ltd. (Tianjin, China). UPLC-grade methanol and acetonitrile were purchased from Merck (Darmstadt, Germany). The 1D and 2D NMR spectra of all isolated compounds were recorded on Bruker DRX-500 spectrometer with the deuterated solvents (DMSO-*d*_6_, CDCl_3_) used as the internal reference, and the chemical shifts are expressed in δ (ppm). HRESIMS data were taken on a Thermo high resolution Q Exactive focus mass spectrometer (Thermo Fisher Scientific, Dreieich, Germany). Dulbecco’s modified Eagle’s medium (DMEM) was obtained from Shanghai VivaCell Biosciences Ltd. Trypsin solution, penicillin-streptomycin mixture, and Fetal bovine serum (FBS) were purchased from Wolway (Weifang, Shandong, China). Methylthiazol-2-yl-2,5-diphenyl tetrazolium bromide (MTT) was obtained from Sigma (Shanghai, China). The 2′,7′-dichlorofluorescin diacetate (DCFH-DA) was acquired from Corning (Corning, NY, USA). Aspartate aminotransferase (AST), Malondialdehyde (MDA), alanine aminotransferase (ALT), superoxide dismutase (SOD), glutathione peroxidase (GSH), and nitric oxide (NO) kits were purchased from Nanjing Jiancheng Biotech Co., Ltd. (Nanjing, China). Paracetamol (APAP) was obtained from Beijing Solaibao (Beijing, China).

### 3.2. Plant Material

The leaves of the *A. fragrans* were collected from Yongde County, Yunnan Province of China (GPS coordinates: 23°45′ N 99°05′ E), and then identified by Dr. Y.P. Liu, Kunming University of Science and Technology. A voucher specimen (No. Cheng20190514-01) was stored in the Faculty of Food Science and Engineering, Kunming University of Science and Technology.

### 3.3. Extraction and Isolation

The air-dried *A. fragrans* leaves were powdered using a grinder. The sample was extracted with 80% methanol aqueous solution using an ultrasonic cleaning bath (200 W) three times (30 min each time). The collected extract solution was evaporated under vacuum using a rotary evaporator (Hei-VAP, Heidolph, Germany) to obtain a crude methanolic extract. The crude extract was then dissolved in distilled water, and further partitioned by ethyl acetate (1:1. *v*/*v*) five times. The ethyl acetate extract from *A. fragrans* leaves (AFEA) was finally obtained by evaporating and lyophilizing the upper solution.

The AFEA (800 g) was eluted with a sequential gradient of CH_2_Cl_2_/methanol (20:1, 10:1, 8:1, 6:1, 4:1, 1:1, and 1:2) by a silica gel column chromatography (CC) to obtain ten fractions (Fr.1–Fr.10). Fr.2 (32 g) was further subjected to a silica gel column with petroleum ether (PE)/acetone solution (20:1, 10:1, and 6:1) to give compounds **1** (17 mg) and **2** (745 mg). Fr.4 (11 g) was then separated to a silica gel column elution with PE/acetone (10:1) to yield compounds **3** (20 mg) and **6** (197 mg). Compounds **4** (279 mg) and **5** (355 mg) were obtained from the Fr.5 (27 g), which was fractionated using Sephadex LH-20 (MeOH) and a silica gel column (PE/acetone, 8:1→5:1). The C_18_ reverse phase column with the eluent of MeOH: H_2_O (20:80 → 80:20) and a silica gel column (PE/acetone, 5:1) were used to purify the Fr.7 (57g) to yield compounds **8** (743 mg), **9** (200 mg), and **16** (18 g). Fr.8 (93g) was separated on a C_18_ reverse phase column with MeOH:H_2_O (40:60→70:30) and further fractionated using a silica gel column (PE/acetone, 8:1 or 5:1) to afford compounds **7** (27 g), **18** (21 mg), and **14** (25 g). Fr. 9 (47g) was separated by a C_18_ reverse phase column with methanol as the eluent, and then prepared and semi-prepared by HPLC with 45% MeOH-H_2_O as the eluent to obtain compounds **13** (85 mg), **17** (17 mg), and **10** (9 mg). Fr.10 (37g) was purified using a C_18_ reverse phase column with the eluent of MeOH:H_2_O (40:60 → 50:50) and then fractionated by the preparative HPLC with CH_3_CN/H_2_O (40:60) to yield compounds **11** (24 mg), **12** (454 mg), and **15** (10 mg).

### 3.4. Hepatoprotective Assessment on HepG2 Cells Induced by APAP

#### 3.4.1. The Assessment of Viability on HepG2 Cells

The HepG2 cells were acquired from the Cell Bank of the Chinese Academy of Sciences (Kunming, China). The cells were cultured in (DMEM), which contained 10% FBS and 1% antibiotic mixture of penicillin and streptomycin (100 mg/mL). The cells were then stored in a 37 °C incubator containing 5% CO_2_ and 95% air.

The cytotoxicity of isolated compounds on HepG2 cells were detected using MTT assay which was slightly modified [9]. Briefly, a 96-well plate was used to culture the HepG2 cells at a density of 1 × 10^5^ cells/mL. After 24 h culture, different diluted compounds concentrations (50/100/150 μM) were added into cells for 20 h of incubation. The MTT solution (0.5 mg/mL) was then added to the cells for 4 h. After removing the MTT solution from each well, dimethyl sulfoxide (DMSO) (200 μL) was added to solubilize the purple formazan crystals. Finally, a SpectraMax M5 microplate reader was applied to measure the absorbance at 570 nm.

HepG2 cell injury was induced with APAP according to the previously reported method [3]. Briefly, the HepG2 cells were incubated in compounds with non-toxic dose and the positive drug N-Acetylcysteine (NAC) (150 μM) for 20 h. After that, 10 mM APAP was incubated with the cells for 20 h. Cell viability was detected to determine the protective effects of isolated compounds on APAP-induced HepG2 cells.

#### 3.4.2. Determination of Inhibitory Effects on AST and ALT

HepG2 cells with a 1.5 × 10^5^ cells/well concentration were seeded in a 6-well plate for 24 h. Afterwards, the cells were cultured with the test compounds. After 20 h, the APAP (10 mM) was then cultured with the cells for 20 h. After that, a 1.5 mL centrifuge tube was used to collect the cells. Then, the cells were washed three times using the pre-cooled PBS, and centrifuged for 10 min at 2500× *g*. The cell supernatants were collected to determine the aspartate aminotransferase (AST) and alanine aminotransferase (ALT) levels.

#### 3.4.3. Cellular Apoptosis Determination

The annexin V-FITC/PI apoptosis kit (Beijing Sizhengbai Biotech Co., Ltd., Beijing, China) was used to detect the HepG2 cell apoptosis [10]. HepG2 cells were pre-cultivated for 20 h with or without the test compounds, and then incubated with APAP at a concentration of 10 mM for another 20 h. The cells were collected, washed with pre-cooled PBS, and resuspended in 100 μL of binding buffer. The cells were then cultured with the annexin V-FITC (10 μL) for 10 min in the dark and stained with propidium iodide (PI) (5 μL) for 5 min in an ice bath. By using the flow cytometry (Guava^®^ easyCyte 6-2L, Millipore, Billerica, MA, USA), cell apoptosis was detected.

#### 3.4.4. Determination of the Generation of Intracellular ROS

The level of intracellular ROS in HepG2 cells induced via APAP was measured using a method previously described [55]. In brief, HepG2 cells were inoculated at a density of 1.5 × 10^5^ cells/mL into a 6-well plate for 24 h pre-incubation, and then treated with the test compounds (150 μM) and APAP (10 mM) for 20 h, respectively. The cells were digested and added with DCFH-DA at a 10 μM concentration for 0.5 h at 37 °C. The flow cytometry was used to record the fluorescence.

#### 3.4.5. Inhibitory Effects on Oxidative Stress

The cell culture process was performed as in Section 3.4.2. After that, the commercial assay kits were applied to determine the MDA, SOD, GSH, and CAT levels based on the manufacturer’s instructions (Nanjing Jiancheng Biotechnology Co., Ltd., Nanjing, China).

### 3.5. Determination of Inflammatory Cytokines on RAW264.7 Cells Induced by LPS

The RAW264.7 cells were acquired from the Cell Bank, Chinese Academy of Sciences (Kunming, China). MTT test was carried out as in Section 3.4.1 to evaluate the cytotoxicity of test compounds. By using a SpectraMax M5 microplate reader, the absorbance was measured at 570 nm. In the subsequent experiments, the maximum nontoxic dose of the test compounds was selected for further experiments.

The RAW 264.7 cells were co-cultured with the test compounds for 2 h in a 12-well plate (1 × 10^5^ cells/well), and then incubated for 24 h with LPS (1 μg/mL). Dexamethasone (DXM) (20 μM) was used as a positive control. Finally, the supernatants were collected, and then the nitric oxide (NO), and pro-inflammatory cytokines, including interleukin-6 (IL-6), interleukin-1β (IL-1β), and tumor necrosis factor-α (TNF-α) levels, were determined using NO kits (Nanjing Jiancheng Biotechnology Co., Ltd.) and enzyme-linked immunosorbent assay (ELISA) kits based on the manufacturer’s instructions, respectively.

### 3.6. Statistical Analysis

All experiments were carried out in triplicate, and the testing data were expressed as mean ± standard deviation (SD). The observed data were analyzed using a One-way analysis of variance (one-way ANOVA). The significant differences (*p* < 0.05) were analyzed via Tukey’s procedure. Origin 8.5 software (OriginLab, Northampton, MA, USA) was applied when performed all analyses.

## 4. Conclusions

The phytochemical investigation of *A. fragrans* resulted in the isolation and identification of eighteen known compounds including triterpenoids (**1**–**5**), aliphatic alcohol (**6**), dihydrochalcones (**7**–**11**), chalcone (**12**), flavanols (**13**–**15**), aurone (**16**), phenolic glycoside (**17**) and lignan (**18**). Ten compounds (**1**–**2**, **4**–**6**, **11**–**12**, and **16**–**18**) were first reported in this species. Compounds **7** and **14** could effectively reduce ROS generation, enhance the activities of antioxidant enzymes for inhibiting oxidative stress, and inhibit cell apoptosis in APAP-induced HepG2 cells. Meanwhile, compounds **7**, **13**, and **14** could also significantly reduce the NO, IL-6, IL-1β, and TNF-α levels in LPS induced RAW264.7 cells. Interestingly, phenolic compounds, especially flavonoids, had considerable hepatoprotective and anti-inflammatory effects because of their phenolic hydroxyl groups. Therefore, the phytochemicals from *A. fragrans* had great potential to prevent liver damage via suppressing oxidative stress and inflammatory response.

## Figures and Tables

**Table 1:** The inhibitory activities of isolated compounds on the excessive generation of NO in LPS-induced RAW264.7 cells.

Compounds	NO (μmol/gprot)	Compounds	NO (μmol/gprot)
C	3.17 ± 0.22	C	3.17 ± 0.22
M	13.65 ± 0.62	M	13.65 ± 0.62
P	7.27 ± 0.52	P	7.27 ± 0.52
1	12.01 ± 0.56	10	10.73 ± 0.37 *
2	7.56 ± 0.27 *	11	11.73 ± 0.73
3	12.33 ± 0.37	12	12.54 ± 0.42
4	12.42 ± 0.65	13	7.33 ± 0.52 *
5	11.51 ± 0.73	14	7.76 ± 0.37 *
6	11.71 ± 1.54	15	8.43 ± 0.85 *
7	9.83 ± 0.51 *	16	12.51 ± 0.28
8	12.32 ± 0.61	17	8.58 ± 0.41 *
9	12.73 ± 0.37	18	9.21 ± 0.54 *

* *p* < 0.05 vs. group M (LPS group); C: normal control group; P: dexamethasone (DXM) group.

## Data Availability

All the data are already present in the article.

## Supplementary Materials

The following supporting information can be downloaded at: https://www.mdpi.com/article/10.3390/molecules28145480/s1, Table S1: The cytotoxic effect of compounds on the survival rate of HepG2 cells; Table S2: Effects of isolated compounds on the survival rate of RAW264.7 cells; Figure S1: The HPLC of compound **1**; Figure S2: The HPLC of compound **2**; Figure S3: The HPLC of compound **3**; Figure S4: The HPLC of compound **4**; Figure S5: The HPLC of compound **5**; Figure S6: The HPLC of compound **6**; Figure S7: The HPLC of compound **7**; Figure S8: The HPLC of compound **8**; Figure S9: The HPLC of compound **9**; Figure S10: The HPLC of compound **10**; Figure S11: The HPLC of compound **11**; Figure S12: The HPLC of compound **12**; Figure S13: The HPLC of compound **13**; Figure S14: The HPLC of compound **14**; Figure S15: The HPLC of compound **15**; Figure S16: The HPLC of compound **16**; Figure S17: The HPLC of compound **17**; Figure S18: The HPLC of compound **18**; The ^13^C NMR data of compounds **1**–**18**.

## Sample Availability

Samples of the compounds are available from the authors.
