# Supplementary material for: Phytochemicals from Anneslea fragrans Wall. and Their Hepatoprotective and Anti-Inflammatory Activities
Source: Molecules. 2023 Jul 18;28(14):5480. doi: 10.3390/molecules28145480 (PMC10384535; doi:10.3390/molecules28145480)

Phytochemicals from *Anneslea fragrans* Wall. and Their Hepatoprotective and Anti-Inflammatory Activities

Yan Wang [1,†], Changshu Cheng [1,†], Tianrui Zhao [1], Jianxin Cao [1], Yaping Liu [1], Yudan Wang [2], Wenbing Zhou [3] and Guiguang Cheng [1,*]

1 Faculty of Food Science and Engineering, Kunming University of Science and Technology, Kunming 650500, China; 20212114040@stu.kust.edu.cn (Y.W.); chengchangshu0409@163.com (C.C.); food363@163.com (T.Z.); jxcao321@hotmail.com (J.C.); liuyaping@kust.edu.cn (Y.L.)

2 Key Laboratory of Chemistry in Ethnic Medicinal Resources, State Ethnic Affairs Commission and Ministry of Education, Yunnan Minzu University, Kunming 650500, China; sdlcwyd@163.com

3 Yunnan Tobacco Company, Yuxi Branch, Yuxi 653100, China; yaowufx2001@163.com

* Correspondence: ggcheng@kmust.edu.cn

† These authors contributed equally to this work.

Contents of Supporting Information

No	Contents	Page
1	Figure S1: The HPLC of compound **1**	3
2	Figure S2: The HPLC of compound **2**	4
3	Figure S3: The HPLC of compound **3**	5
4	Figure S4: The HPLC of compound **4**	6
5	Figure S5: The HPLC of compound **5**	7
6	Figure S6: The HPLC of compound **6**	8
7	Figure S7: The HPLC of compound **7**	9
8	Figure S8: The HPLC of compound **8**	10
9	Figure S9: The HPLC of compound **9**	11
10	Figure S10: The HPLC of compound **10**	12
11	Figure S11: The HPLC of compound **11**	13
12	Figure S12: The HPLC of compound **12**	14
13	Figure S13: The HPLC of compound **13**	15
14	Figure S14: The HPLC of compound **14**	16
15	Figure S15: The HPLC of compound **15**	17
16	Figure S16: The HPLC of compound **16**	18
17	Figure S17: The HPLC of compound **17**	19
18	Figure S18: The HPLC of compound **18**	20

Figure S1. The HPLC of compound **1**.

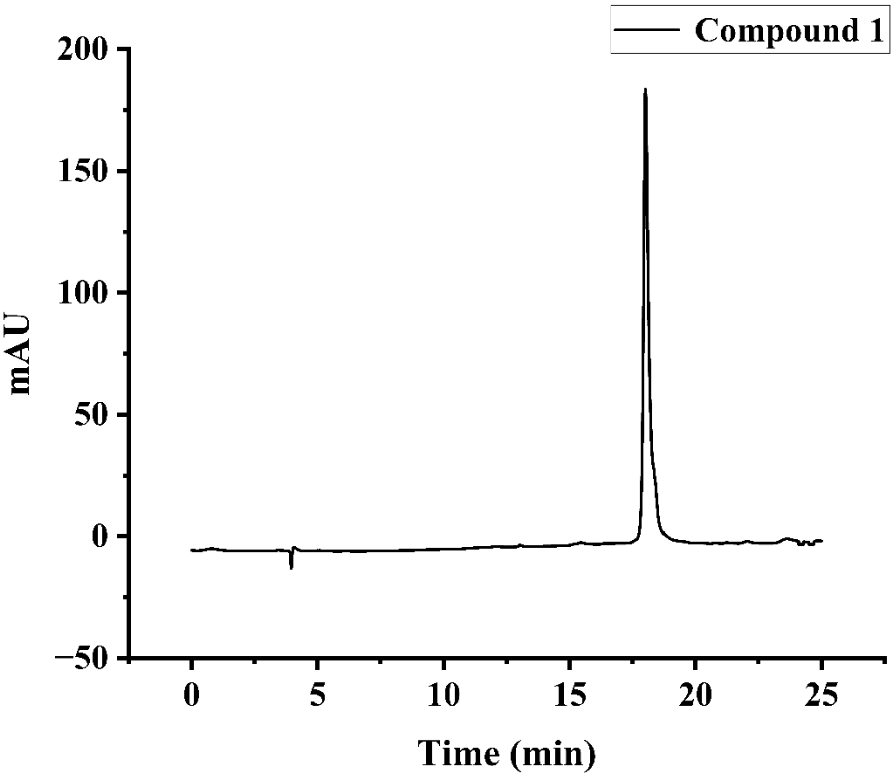

Figure S2. The HPLC of compound **2**.

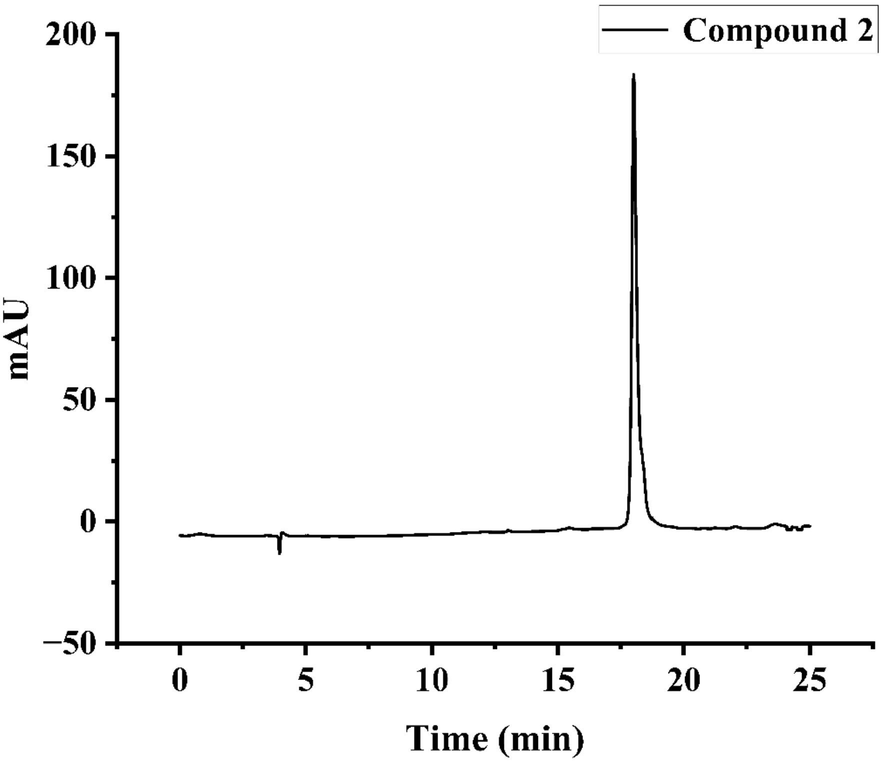

Figure S3. The HPLC of compound **3**.

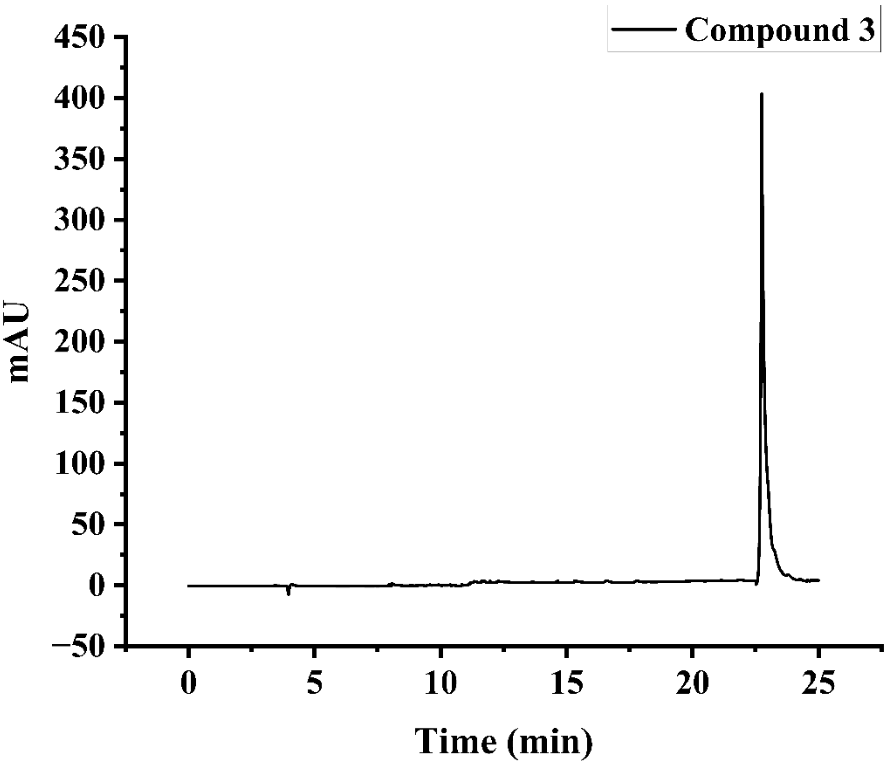

Figure S4. The HPLC of compound **4**.

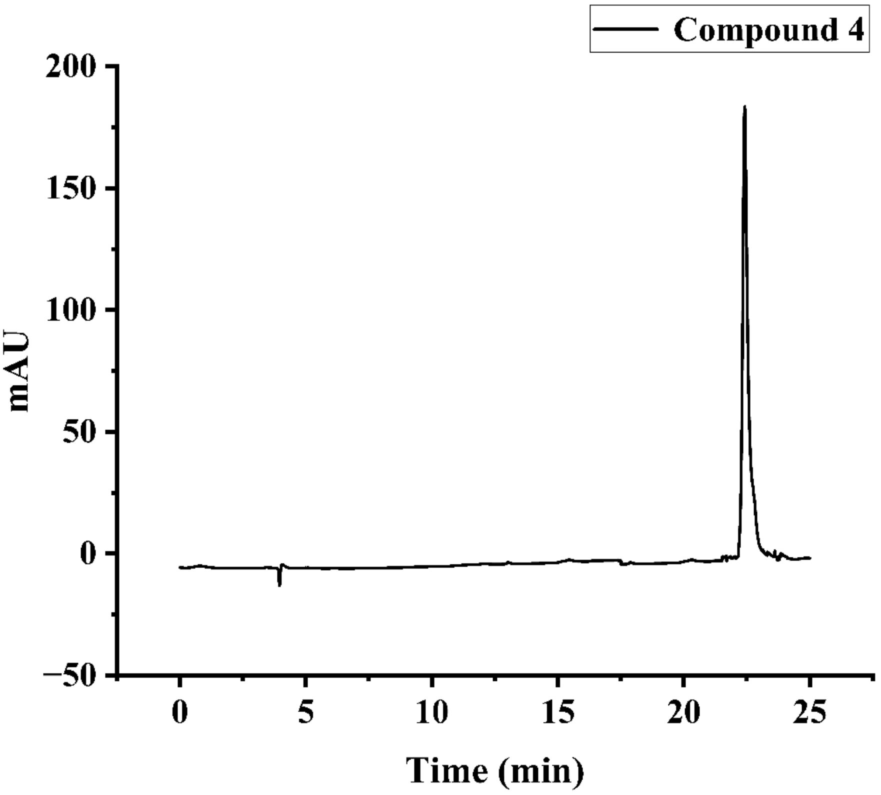

Figure S5. The HPLC of compound **5**.

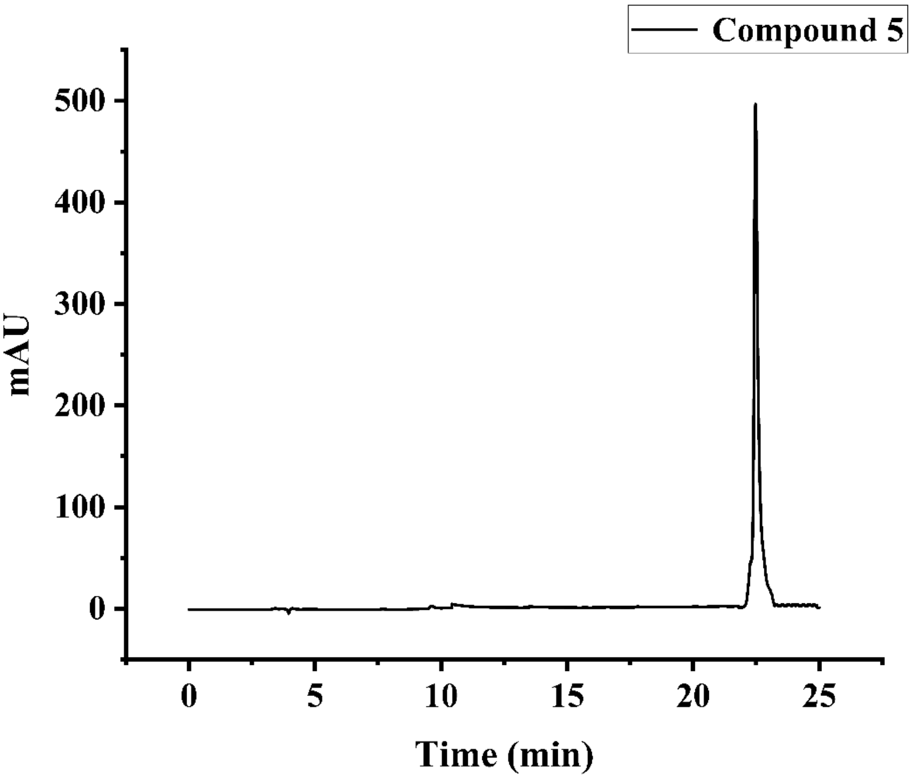

Figure S6. The HPLC of compound **6**.

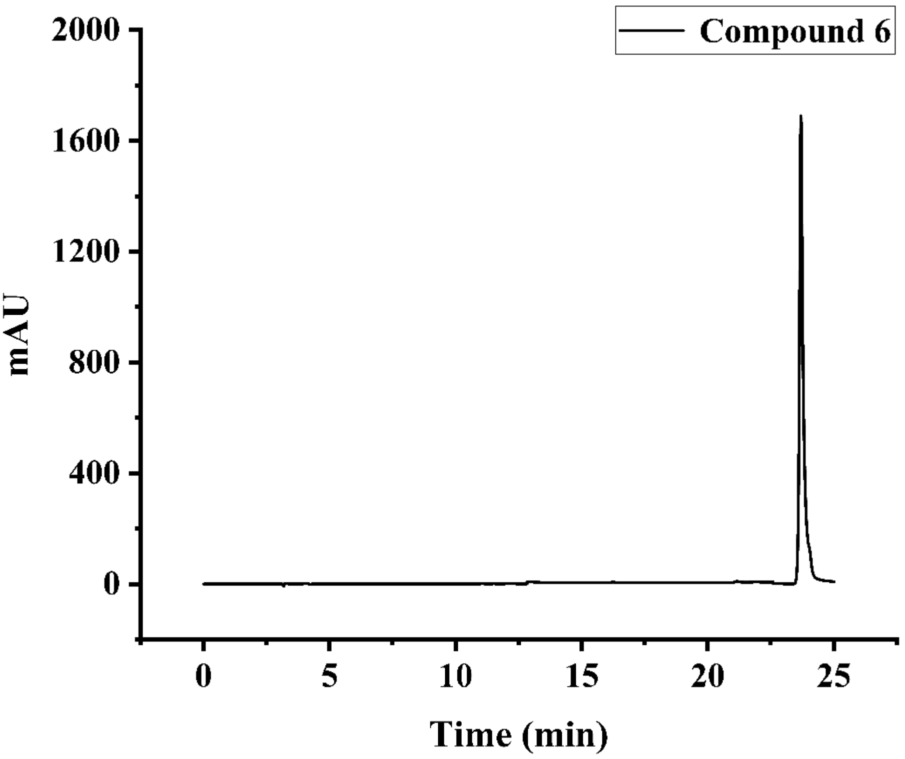

Figure S7. The HPLC of compound **7**.

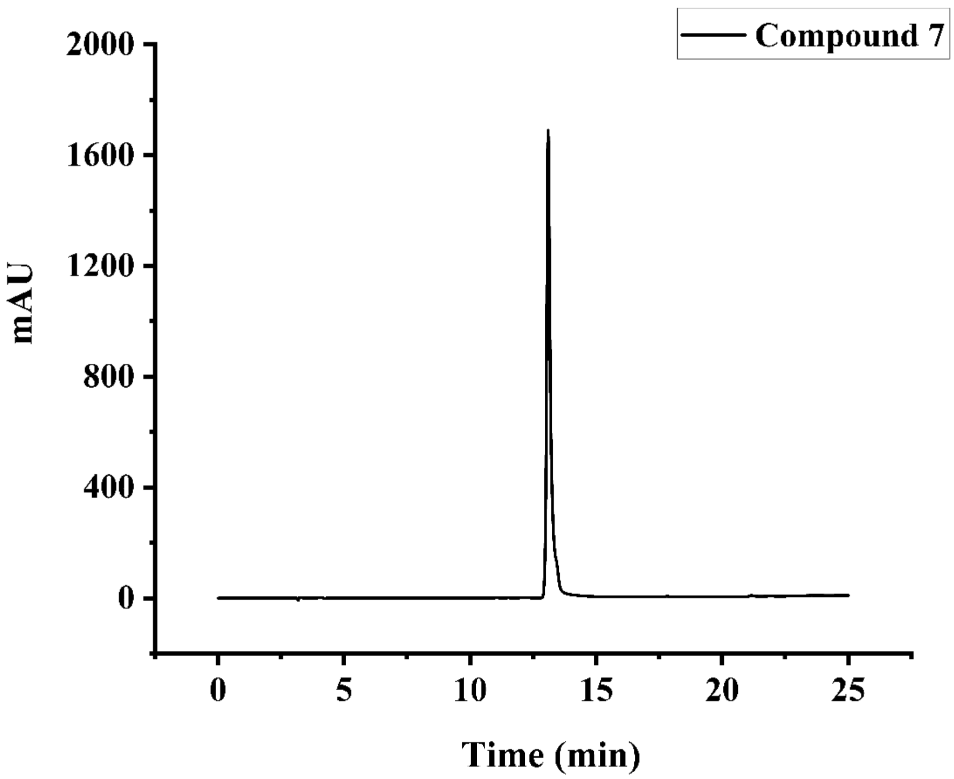

Figure S8. The HPLC of compound **8**.

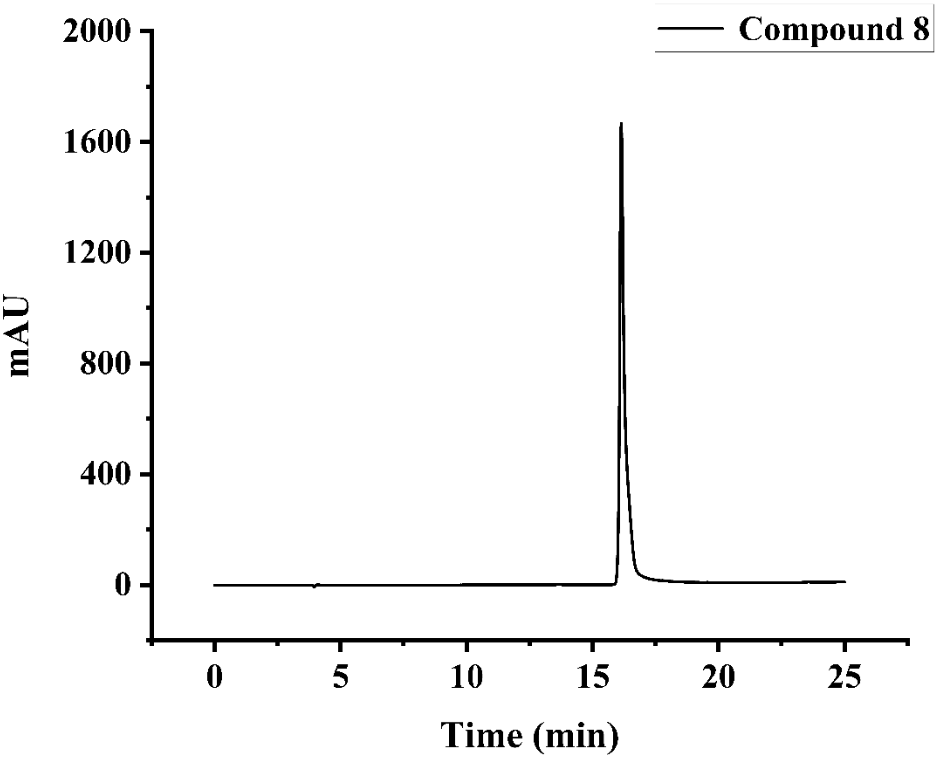

Figure S9. The HPLC of compound **9**.

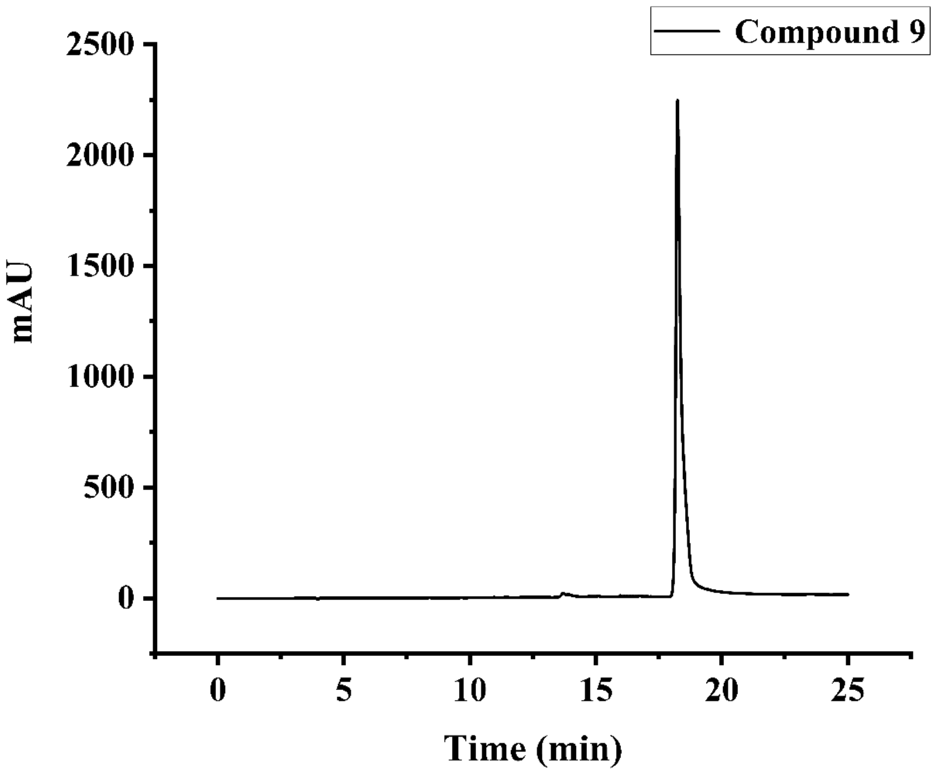

Figure S10. The HPLC of compound **10**.

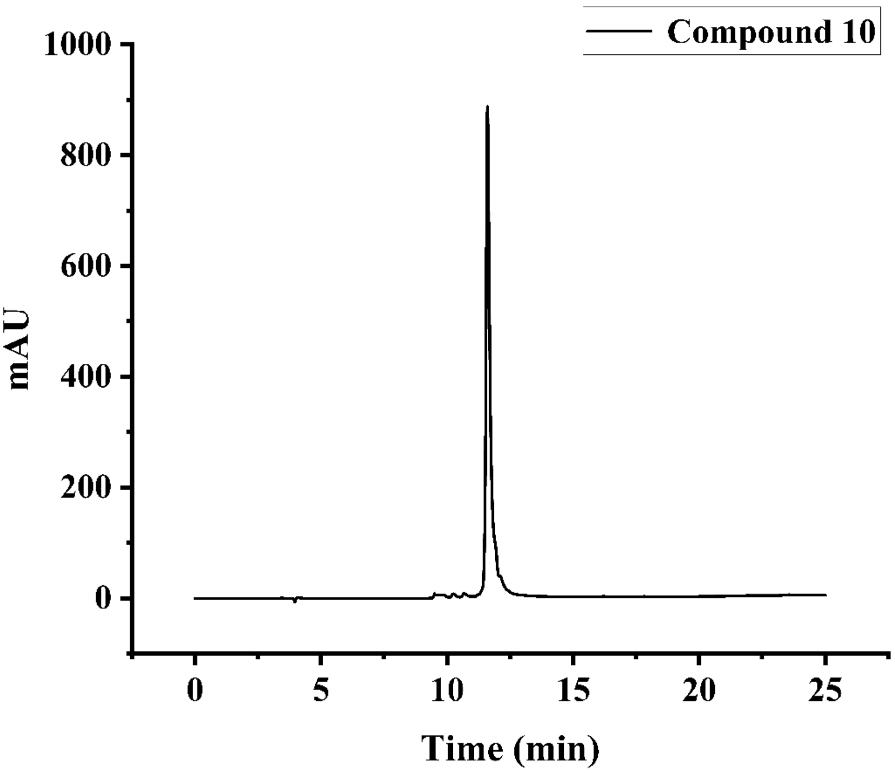

Figure S11. The HPLC of compound **11**.

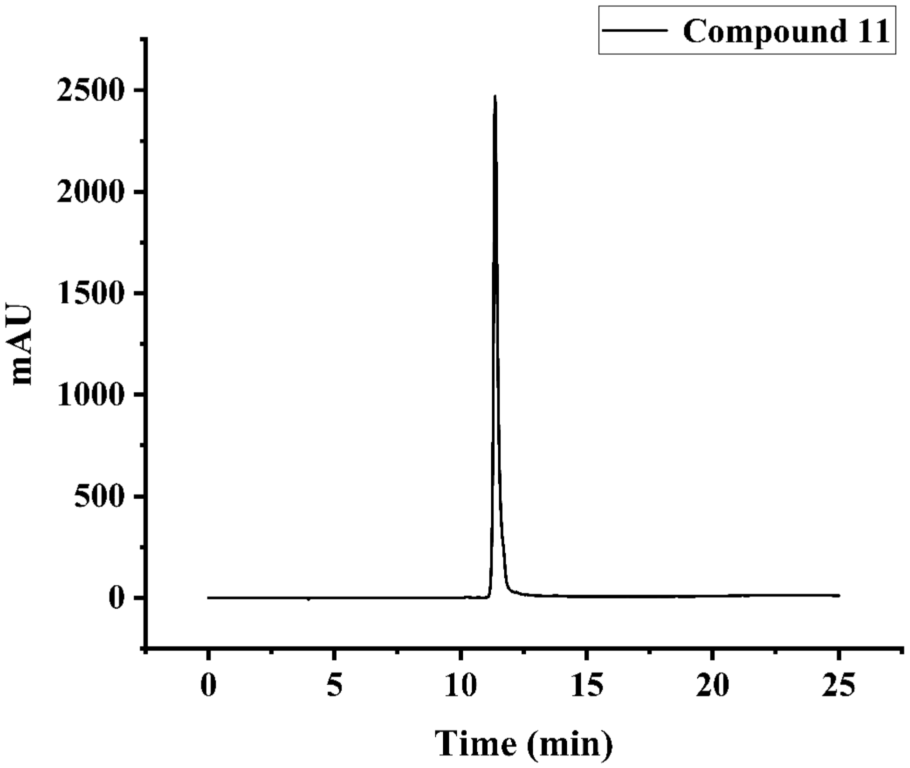

Figure S12. The HPLC of compound **12**.

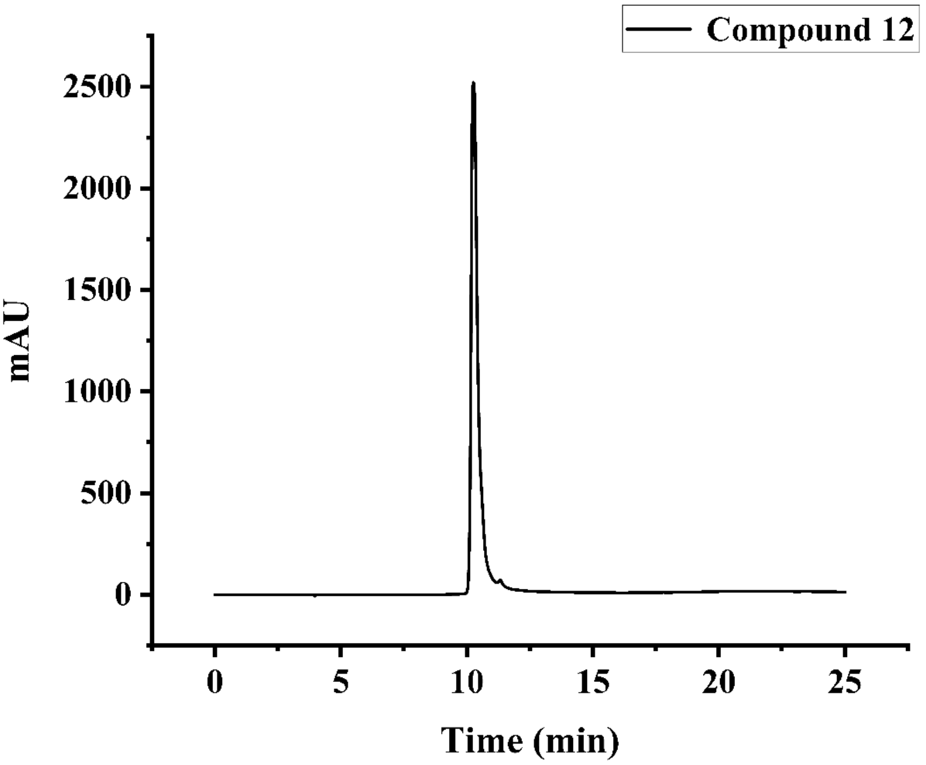

Figure S13. The HPLC of compound **13**.

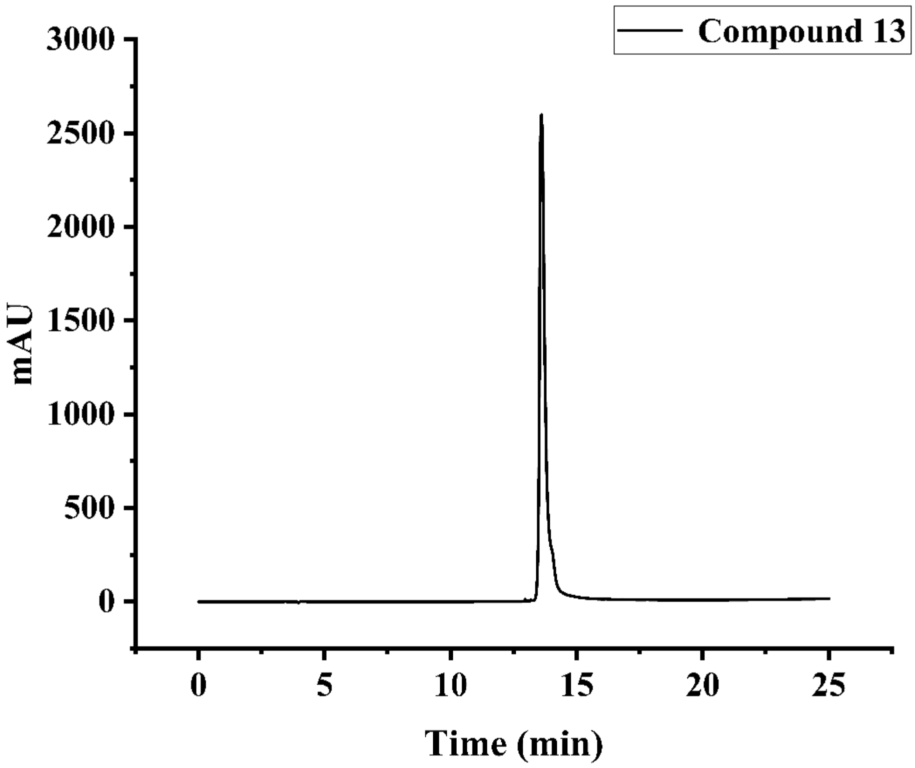

Figure S14. The HPLC of compound **14**.

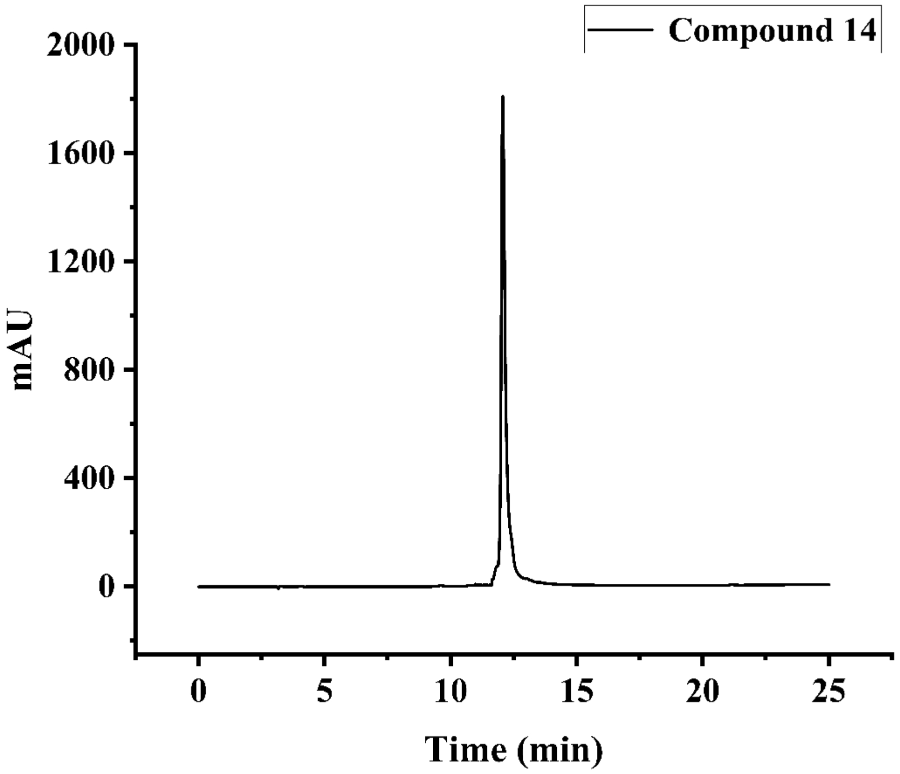

Figure S15. The HPLC of compound **15**.

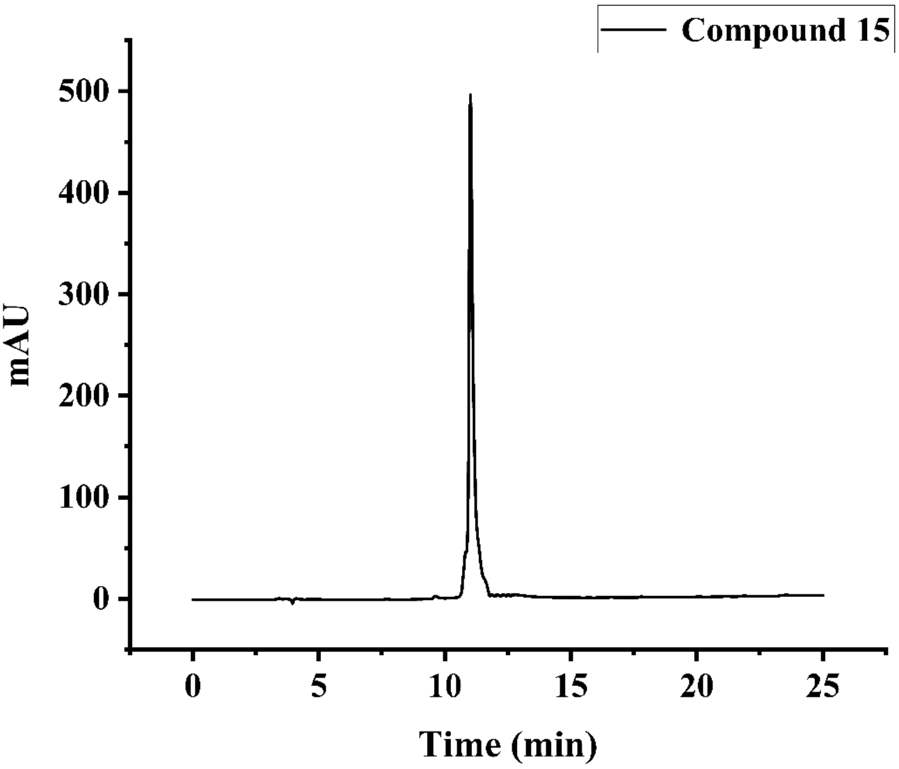

Figure S16. The HPLC of compound **16**.

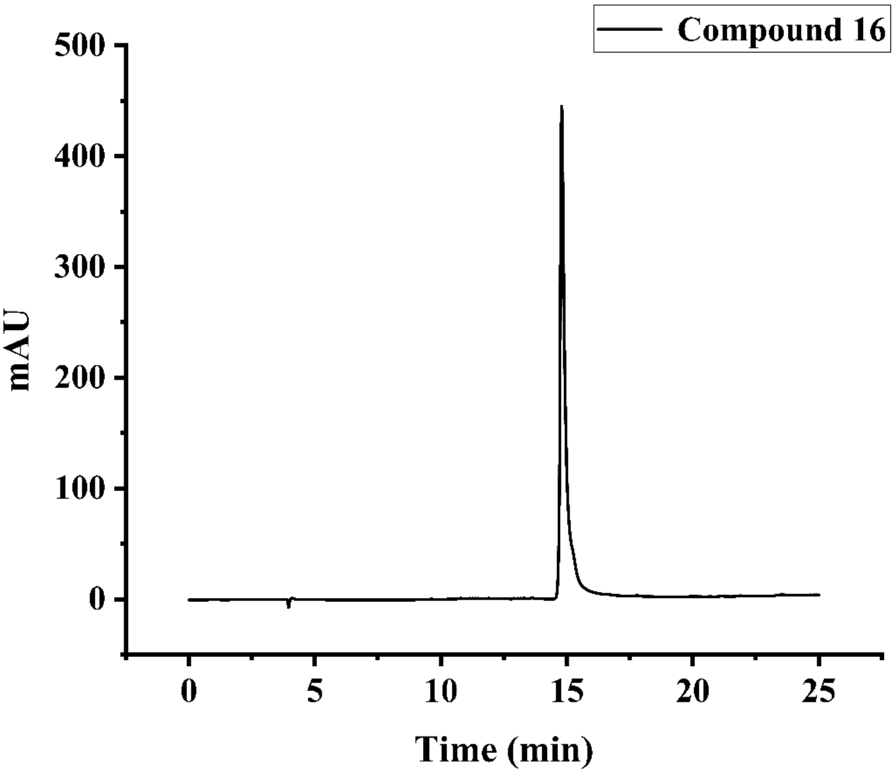

Figure S17. The HPLC of compound **17**.

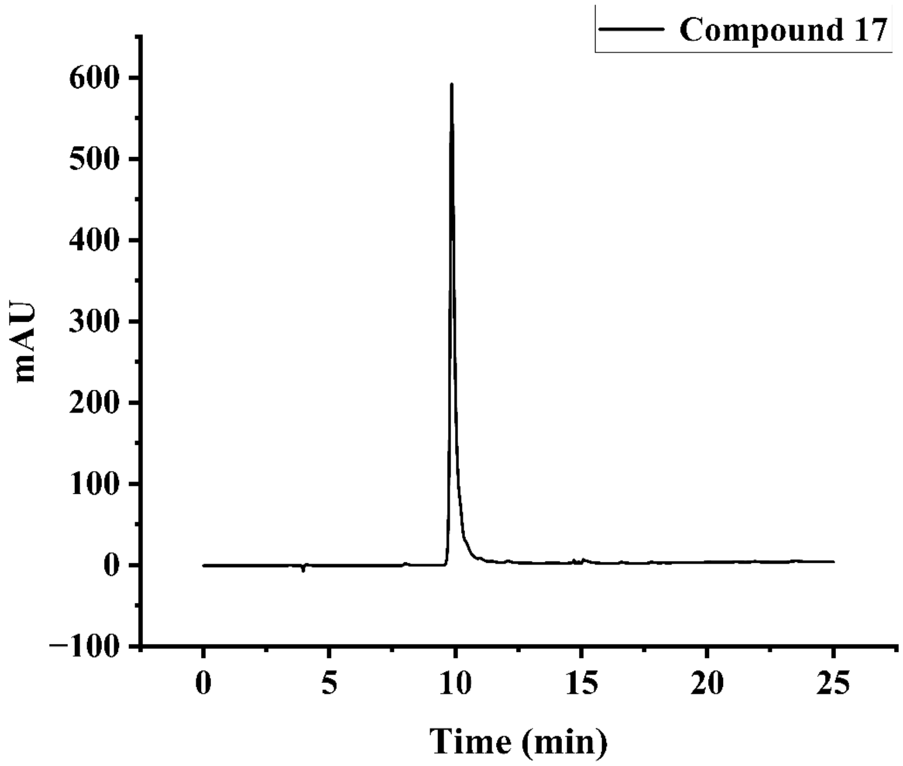

Figure S18. The HPLC of compound **18**.

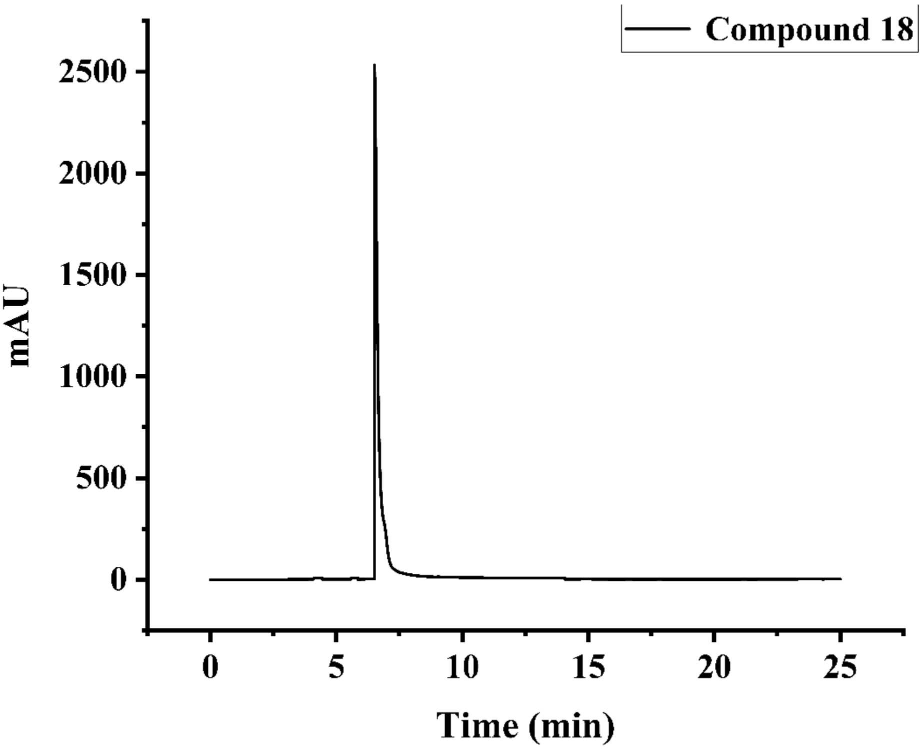

Supplement: Supplementary file 1 [file molecules-28-05480-s001.zip › Supplementary Figure.pdf]
