# Supplementary material for: Phytochemicals from Anneslea fragrans Wall. and Their Hepatoprotective and Anti-Inflammatory Activities
Source: Molecules. 2023 Jul 18;28(14):5480. doi: 10.3390/molecules28145480 (PMC10384535; doi:10.3390/molecules28145480)
Supplement: Supplementary file 1 [file molecules-28-05480-s001.zip › Supplementary Materials.docx.pdf]

Phytochemicals from *Anneslea fragrans* Wall. and Their Hepatoprotective and Anti-Inflammatory Activities

Yan Wang [1,†], Changshu Cheng [1,†], Tianrui Zhao [1], Jianxin Cao [1], Yaping Liu [1], Yudan Wang [2], Wenbing Zhou [3] and Guiguang Cheng [1,*]

1 Faculty of Food Science and Engineering, Kunming University of Science and Technology, Kunming 650500, China; 20212114040@stu.kust.edu.cn (Y.W.); chengchangshu0409@163.com (C.C.); food363@163.com (T.Z.); jxcao321@hotmail.com (J.C.); liuyaping@kust.edu.cn (Y.L.)

2 Key Laboratory of Chemistry in Ethnic Medicinal Resources, State Ethnic Affairs Commission and Ministry of Education, Yunnan Minzu University, Kunming 650500, China; sdlcwyd@163.com

3 Yunnan Tobacco Company, Yuxi Branch, Yuxi 653100, China; yaowufx2001@163.com

* Correspondence: ggcheng@kmust.edu.cn

† These authors contributed equally to this work.

The ^{13}C NMR data of compounds 1-18

Compound **1**: White powder, $C_{30}H_{50}O$, HR-ESI-MS *m/z:* 427.3937 $[M+H]^+$. ^{13}C NMR (125 MHz, $CDCl_3$): δc 141.5 (C-5), 122.1 (C-6), 77.3 (C-3), 49.6 (C-10), 47.4 (C-8), 43.0 (C-18), 40.8 (C-4), 39.2 (C-14), 38.9 (C-16), 37.8 (C-13), 36.0 (C-22), 35.0 (C-21), 34.8 (C-9), 34.6 (C-11), 34.5 (C-29), 33.0 (C-19), 32.4 (C-30), 32.0 (C-15,28), 30.3 (C-12), 30.0 (C-17), 28.9 (C-23), 28.2 (C-20), 27.8(C-2), 25.4 (C-24), 23.6 (C-7), 19.6 (C-27), 18.4 (C-26), 18.2 (C-1), 16.2 (C-25).

Compound **2**: White powder, $C_{30}H_{50}O$, HR-ESI-MS *m/z*: 427.3937 $[M+H]^+$. ^{13}C NMR (125 MHz, $CDCl_3$): δc 213.4 (C-3), 59.4 (C-5), 58.2 (C-9), 53.1 (C-6), 42.7 (C-8), 42.2 (C-18), 41.5 (C-14), 41.2 (C-13), 39.6 (C-10), 39.2 (C-4), 38.2 (C-1), 37.4 (C-22), 35.9 (C-19), 35.6 (C-29), 35.3 (C-11), 35.0 (C-30), 32.7 (C-17), 32.4 (C-21), 32.2 (C-15), 31.8 (C-12), 30.9 (C-16), 30.5 (C-20), 30.0 (C-23), 28.1 (C-2), 22.3 (C-24), 20.2 (C-28), 18.7 (C-27), 18.2 (C-25), 17.9 (C-7), 14.6 (C-26).

Compound **3:** White powder, $C_{30}H_{50}O$, HR-ESI-MS *m/z*: 425.3770 $[M-H]^+$. ^{13}C NMR (125 MHz, $CDCl_3$): δc 150.4 (C-20), 109.7 (C-29), 78.9 (C-3), 60.5 (C-28), 55.2 (C-5), 50.4 (C-9), 48.7 (C-18), 47.7 (C-17, C-19), 42.7 (C-14), 40.9 (C-8), 38.8 (C-4), 38.7 (C-1), 37.3 (C-13), 37.1 (C-10), 34.2 (C-7), 33.9 (C-22), 29.7 (C-21), 29.1 (C-16), 27.9 (C-23), 27.4 (C-2), 27.0 (C-15), 25.2 (C-12), 20.8 (C-11), 19.0 (C-30), 18.3 (C-6), 16.1 (C-26), 15.9 (C-25), 15.3 (C-24), 14.7 (C-27).

Compound **4:** White needle, $C_{30}H_{48}O_5$, HR-ESI-MS *m/z*: 487.3429 $[M-H]^+$. ^{13}C NMR (125 MHz, $CDCl_3$): δc 178.9 (C-28), 138.6 (C-13), 126.8 (C-12), 77.9 (C-3), 71.6 (C-19), 64.7 (C-2), 53.1 (C-18), 47.6 (C-5), 46.9 (C-9), 46.5 (C-17), 41.6 (C-1), 41.4 (C-14), 41.1 (C-20), 40.4 (C-8), 38.0 (C-4), 37.8 (C-22), 37.3 (C-10), 32.6 (C-7), 28.9 (C-23), 28.0 (C-15), 26.4 (C-21), 25.9 (C-29), 25.1 (C-16), 24.1 (C-27), 23.1 (C-11), 21.8 (C-30), 17.7 (C-6), 16.6 (C-26), 16.3 (C-24), 16.1 (C-25).

Compound **5:** White powder, $C_{30}H_{48}O_4$, HR-ESI-MS *m/z*: 471.3479 $[M-H]^+$. ^{13}C NMR (125 MHz, $CDCl_3$): δc 178.3 (C-28), 138.2 (C-13), 124.5 (C-12), 82.2 (C-3), 67.1 (C-2), 54.7 (C-18), 52.3 (C-5), 47.0 (C-17), 46.9 (C-9), 46.8 (C-1), 41.7 (C-14), 38.5 (C-8), 38.4 (C-19), 37.5 (C-20), 36.3 (C-22), 32.6 (C-4), 30.2 (C-10), 29.8 (C-23), 28.8 (C-7), 27.5 (C-21), 23.8 (C-15), 23.2 (C-27), 22.9 (C-16), 21.8 (C-30), 21.0 (C-11), 18.0 (C-6,30), 17.2 (C-26), 17.0 (C-29), 16.9 (C-25), 16.5 (C-24).

Compound **6:** White powder, $C_{31}H_{64}O$, HR-ESI-MS *m/z*: 453.3428 $[M+H]^+$. ^{13}C NMR (125 MHz, $CDCl_3$): δc 63.1 (1-CH_2OH), 22.6-32.8 (2~30-CH_2), 14.1 (31-CH_3).

Compound **7:** Yellow needle crystal, $C_{21}H_{24}O_9$, HR-ESI-MS *m/z*: 419.1348 [M-H]$^+$. ^{13}C NMR (125 MHz, DMSO-d_6): δc 204.4 (C-7′), 163.5 (C-2′), 163.3 (C-4′), 155.4 (C-4), 132.6 (C-6′), 130.9 (C-1), 129.2 (C-2,6), 115.0 (C-3,5), 114.4 (C-1′), 108.3 (C-5′), 103.4 (C-1″), 99.6 (C-3′), 77.1 (C-5″), 76.4 (C-3″), 73.0 (C-2″), 69.5 (C-4″), 62.3 (C-6″), 39.9 (C-α), 28.9 (C-β).

Compound **8:** Yellow oil, $C_{15}H_{14}O_5$, HR-ESI-MS *m/z*: 274.0769 [M]. ^{13}C NMR (125 MHz, DMSO-d_6): δc 203.9 (C-7′), 164.7 (C-2′), 164.2 (C-4′), 145.0 (C-3), 143.3 (C-4), 133.1 (C-6′), 131.7 (C-1), 118.9 (C-6), 115.8 (C-2), 115.4 (C-5), 112.5 (C-1′), 108.1 (C-5′), 102.4 (C-3′), 39.5 (C-α), 29.2 (C-β).

Compound **9:** Yellow crystal, $C_{15}H_{14}O_4$, HR-ESI-MS *m/z*: 258.0818 [M]. ^{13}C NMR (125 MHz, DMSO-d_6): δc 203.8 (C-7′), 164.8 (C-2′), 164.2 (C-4′), 155.5 (C-4), 133.1 (C-6′), 131.0 (C-1), 129.2 (C-2,6), 115.1 (C-3,5), 112.5 (C-1′), 108.1 (C-5′), 102.4 (C-3′), 38.6 (C-α), 29.1(C-β).

Compound **10:** Yellow powder, $C_{22}H_{26}O_{10}$, HR-ESI-MS *m/z*: 449.1463 [M-H]$^+$. ^{13}C NMR (125 MHz, DMSO-d_6): δ_C 198.8 (C-7′), 162.6 (C-4′), 158.7 (C-2′), 147.3 (C-3), 144.3 (C-4), 132.5 (C-1), 131.7 (C-6′), 120.3 (C-1′), 119.6 (C-6), 115.2 (C-5), 112.3 (C-2), 109.3 (C-5′), 102.0 (C-3′), 100.6 (C-1″), 77.1 (C-3″), 76.6 (C-5″), 73.4 (C-2″), 69.5 (C-4″), 62.7 (C-6″), 55.5 (3-OCH_3), 44.6 (C-α), 29.6 (C-β).

Compound **11**: White crystals, $C_{22}H_{26}O_{10}$, HR-ESI-MS *m/z*: 451.1885 [M+H]$^+$. ^{13}C NMR (125 MHz, DMSO-d_6): δc 204.5 (C-7′), 163.5 (C-2′), 163.3 (C-4′), 147.4 (C-3), 144.6 (C-4), 132.6 (C-6′), 131.6 (C-1), 120.4 (C-6), 115.2 (C-5), 114.5 (C-1′), 112.6 (C-2), 108.3 (C-5′), 103.3 (C-1″), 99.5 (C-3′), 77.0 (C-5″), 76.3 (C-3″), 73.0 (C-2″), 69.5 (C-4″), 60.5 (C-6″), 55.5 (3-OCH_3), 39.5 (C-α), 29.4 (C-β).

Compound **12**: Yellow crystal, $C_{21}H_{22}O_{10}$, HR-ESI-MS *m/z*: 433.1139 [M-H]$^+$. ^{13}C NMR (125 MHz, DMSO-d_6): δc 192.0 (C-7′), 165.0 (C-2′), 163.5 (C-4′), 149.1 (C-3), 145.6 (C-α), 145.5 (C-4), 132.4 (C-6′), 126.1 (C-1), 122.6 (C-6), 117.3 (C-β), 116.0 (C-5), 115.7 (C-2), 114.8 (C-1′), 108.2 (C-5′), 103.4 (C-3′), 101.6 (C-1″), 77.1 (C-3″), 76.4 (C-5″), 73.1 (C-2″), 69.6 (C-4″), 60.6 (C-6″).

Compound **13**: White powder, $C_{21}H_{20}O_{11}$, HR-ESI-MS *m/z*: 447.2132 [M-H]$^+$. ^{13}C NMR (125 MHz, DMSO-d_6): δ_C 178.2 (C-4), 166.4 (C-7), 161.8 (C-5), 158.8 (C-4′), 157.7 (C-9), 148.4 (C-2), 135.1 (C-3), 129.2 (C-2′,6′), 122.9 (C-1′), 115.8 (C-3′,5′), 109.6 (C-6), 102.3 (C-10), 94.0 (C-8), 81.6 (C-5″), 78.9 (C-3″), 75.7 (C-1″), 74.1 (C-2″), 71.5 (C-4″), 62.2 (C-6″).

Compound **14:** Yellow oil, $C_{21}H_{20}O_{11}$, HR-ESI-MS *m/z*: 447.0933 $[M-H]^+$. ^{13}C NMR (125 MHz, DMSO-d_6): δ_C 177.7 (C-4), 164.2 (C-7), 161.3 (C-5), 157.3 (C-9), 156.4 (C-2), 148.4 (C-4′), 145.2 (C-3′), 134.2 (C-3), 121.1 (C-6′), 120.7 (C-1′), 115.6 (C-5′), 115.4 (C-2′), 104 (C-10), 101.8 (C-1″), 98.7 (C-6), 93.6 (C-8), 71.1 (C-2″), 70.6 (C-3″), 70.3 (C-4″), 70.1 (C-5″), 17.5 (C-6″).

Compound **15**: Yellow powder, $C_{21}H_{20}O_{12}$, HR-ESI-MS *m/z*: 463.0897 $[M-H]^+$. ^{13}C NMR (125 MHz, DMSO-d_6): δ_C 177.6 (C-4), 164.1 (C-7), 161.2 (C-5), 156.3 (C-2), 156.2 (C-9), 148.5 (C-4′), 144.8 (C-3′), 133.4 (C-3), 122.1 (C-6′), 121.1 (C-1′), 115.9 (C-5′), 115.1 (C-2′), 103.8 (C-10), 101.8 (C-1″), 98.7 (C-6), 93.5 (C-8), 75.8 (C-5″), 73.1 (C-3″), 71.2 (C-2″), 67.9 (C-4″), 60.1 (C-6″).

Compound **16**: Yellow powder, $C_{15}H_{10}O_5$, HR-ESI-MS *m/z*: 269.0455 $[M-H]^+$. ^{13}C NMR (125 MHz, DMSO-d_6): δ_C 181.2 (C-3), 167.4 (C-6), 166.1 (C-8), 148.1 (C-4′), 148.6 (C-2), 145.5 (C-3′), 125.7 (C-4), 124.5 (C-6′), 123.4 (C-1′), 117.9 (C-2′), 116.5 (C-5′), 116.1 (C-9), 114.1 (C-10), 113.9 (C-7), 98.3 (C-5).

Compound **17**: Yellow powder, $C_{15}H_{20}O_8$, HR-ESI-MS *m/z*: 327.1139 $[M-H]^+$. ^{13}C NMR (125 MHz, DMSO-d_6): δ_C 203.3 (C-7), 163.5 (C-2), 163.4 (C-4), 132.8 (C-6), 121.7 (C-1), 108.3 (C-5), 103.4 (C-3), 99.6 (C-1′), 77.1 (C-3′), 76.4 (C-5′), 73.1 (C-2′), 69.5 (C-4′), 67.4 (C-6′), 58.1 (4-OCH_3), 38.1 (C-8).

Compound **18**: White powder, $C_{31}H_{38}O_{11}$, HR-ESI-MS *m/z*: 585.2344 $[M-H]^+$. ^{13}C NMR (125 MHz, DMSO-d_6): δ_C 152.6 (C-3′,C-5′), 146.8 (C-3″), 145.4 (C-4″), 145.3 (C-3), 147.4 (C-4), 136.9 (C-1), 138.7 (C-1′), 135.3 (C-4′), 132.9 (C-1″), 128.9 (C-5), 119.2 (C-6″), 116.4 (C-6), 114.6 (C-5″), 112.5 (C-2), 111.0 (C-2″), 103.3 (C-2′,6′), 87.1 (C-8″), 86.7 (C-7′), 71.5 (C-7″), 62.8 (C-9′), 60.2 (C-9″), 56.0 (3-OCH_3), 55.7 (3′,5′-OCH_3), 55.5 (3″-OCH_3), 53.3 (C-8′), 34.7 (C-8), 31.5 (C-7), 62.3 (C-9).
