# Supplementary material for: Phytochemicals from Anneslea fragrans Wall. and Their Hepatoprotective and Anti-Inflammatory Activities
Source: Molecules. 2023 Jul 18;28(14):5480. doi: 10.3390/molecules28145480 (PMC10384535; doi:10.3390/molecules28145480)
Supplement: Supplementary file 1 [file molecules-28-05480-s001.zip › Supplementary Table.pdf]

Phytochemicals from *Anneslea fragrans* Wall. and Their Hepatoprotective and Anti-Inflammatory Activities

Yan Wang [1,†], Changshu Cheng [1,†], Tianrui Zhao [1], Jianxin Cao [1], Yaping Liu [1], Yudan Wang [2], Wenbing Zhou [3] and Guiguang Cheng [1,*]

1 Faculty of Food Science and Engineering, Kunming University of Science and Technology, Kunming 650500, China; 20212114040@stu.kust.edu.cn (Y.W.); chengchangshu0409@163.com (C.C.); food363@163.com (T.Z.); jxcao321@hotmail.com (J.C.); liuyaping@kust.edu.cn (Y.L.)

2 Key Laboratory of Chemistry in Ethnic Medicinal Resources, State Ethnic Affairs Commission and Ministry of Education, Yunnan Minzu University, Kunming 650500, China; sdlcwyd@163.com

3 Yunnan Tobacco Company, Yuxi Branch, Yuxi 653100, China; yaowufx2001@163.com

* Correspondence: ggcheng@kmust.edu.cn

† These authors contributed equally to this work.

Contents of Supporting Information

No	Contents	Page
1	Table S1: The cytotoxic effect of compounds on the survival rate of HepG2 cells	3
2	Table S2: Effects of isolated compounds on the survival rate of RAW264.7 cells	4

Table S1. The cytotoxic effect of compounds on the survival rate of HepG2 cells.

**Compounds**	**Concentration(μM)**		
	**50**	**100**	**150**
**1**	96.73 ±3.33	94.33 ± 2.72	92.36 ± 4.27
**2**	113.92 ± 1.14	108.91 ± 3.21	97.54 ± 2.45
**3**	98.66 ± 2.76	94.64 ± 4.48	88.36 ± 2.41
**4**	113.94 ± 5.24	102.15 ± 3.25	96.18 ± 3.64
**5**	99.35 ± 3.26	95.25 ± 4.26	93.23 ± 2.52
**6**	114.11 ± 2.42	107.23 ± 3.54	102.1 ± 1.53
**7**	105.79 ± 3.36	101.04 ± 3.21	96.75 ± 0.74
**8**	113.93 ± 5.44	105.85 ± 2.24	104.37 ± 1.32
**9**	103.72 ± 2.73	91.95 ± 0.66	79.45 ± 0.89
**10**	93.27 ± 1.14	96.18 ± 0.89	93.93 ± 1.70
**11**	103.07 ± 3.13	103.71 ± 1.21	99.61 ± 2.43
**12**	107.13 ± 1.27	108.87 ± 3.20	103.95 ± 1.09
**13**	103.14 ± 1.37	102.44 ± 4.73	98.47 ± 2.14
**14**	105.05 ± 0.68	105.14 ± 1.12	101.54 ± 0.98
**15**	101.15 ± 2.78	101.82 ± 0.96	101.16 ± 1.58
**16**	96.75 ± 2.31	96.34 ± 2.26	98.23 ± 3.16
**17**	93.12 ± 1.36	96.51 ± 1.52	92.27 ± 0.75
**18**	102.37 ± 1.21	102.41 ± 1.88	95.06 ± 0.85

Table S2. Effects of isolated compounds on the survival rate of RAW264.7 cells.

**Compounds**	**Concentration(μM)**		
	**50**	**100**	**200**
**1**	96.73 ± 3.33	96.31 ± 2.72	95.36 ± 2.22
**2**	94.92 ± 1.14	98.91 ± 2.27	97.74 ± 2.42
**3**	98.66 ± 2.76	94.64 ± 3.48	98.36 ± 2.41
**4**	103.94 ± 5.24	98.15 ± 3.25	96.18 ± 2.64
**5**	94.35 ± 3.22	95.47 ± 2.21	94.23 ± 1.98
**6**	94.12 ± 2.45	73.23 ± 1.54	36.12 ± 1.53
**7**	95.79 ± 2.78	101.04 ± 2.24	96.75 ± 0.74
**8**	93.93 ± 3.13	85.85 ± 1.34	70.37 ± 1.62
**9**	98.72 ± 2.73	71.95 ± 1.62	57.32 ± 1.82
**10**	103.92 ± 1.92	101.69 ± 2.11	102.69 ± 3.17
**11**	103.07 ± 4.13	97.71 ± 1.43	97.61 ± 2.43
**12**	107.13 ± 1.27	98.87 ± 3.24	86.59 ± 1.33
**13**	101.05 ± 0.68	98.14 ± 1.12	98.54 ± 1.67
**14**	96.75 ± 2.31	96.34 ± 2.26	98.23 ± 3.16
**15**	93.27 ± 1.14	96.18 ± 2.89	93.93 ± 2.73
**16**	101.77 ± 1.52	98.59 ± 1.76	96.75 ± 0.48
**17**	93.12 ± 1.36	96.51 ± 1.52	92.27 ± 0.75
**18**	102.67 ± 0.85	94.56 ± 1.36	95.83 ± 1.51
